# Comparative efficacy of virtual reality interventions with varying immersion levels on upper limb motor function, hand dexterity, and independence in the daily life of stroke survivors: a systematic review and network meta-analysis

**DOI:** 10.3389/fneur.2026.1797313

**Published:** 2026-09-23

**Authors:** Haoyuan Li, Chunhao Dai, Ziting Xu, Teng Yang, Wen Li, Luyao Yan, Lijuan Yang, Minmin Leng

**Affiliations:** Department of Nursing, Shandong Provincial Hospital Affiliated to Shandong First Medical University, Jinan, Shandong, China

**Keywords:** network meta-analysis, stroke, systematic review, upper limb motor function, virtual reality

## Abstract

**Background:**

Upper limb motor function impairment is a highly prevalent sequela following stroke, significantly compromising patients’ independence in daily life and decreasing their quality of life. Virtual reality represents a promising therapeutic intervention for this condition; however, the comparative efficacy of virtual reality interventions with different levels of immersion remains unclear.

**Methods:**

A comprehensive electronic literature search was performed in PubMed, Embase, the Cochrane Library, Web of Science, and CINAHL up to July 2025. Randomized controlled trials (RCTs) evaluating the use of different levels of immersion virtual reality intervention (such as immersive virtual reality, semi-immersive virtual reality and nonimmersive virtual reality) compared with routine rehabilitation for stroke with upper limb motor function impairment were included. A random-effects model was used for the network meta-analysis. Efficacy was assessed via standardized mean differences (SMDs) with 95% confidence intervals (CIs), and interventions were ranked via surface under the cumulative ranking curve (SUCRA) probabilities.

**Results:**

Twenty-nine RCTs involving 1,524 participants were included from 6,830 records. Compared with the control interventions, the virtual reality intervention improved upper limb motor function, enhanced hand dexterity, and enhanced independence in the daily life of stroke patients. With respect to upper limb motor function, immersive virtual reality was ranked the highest (SUCRA = 86.10%; SMD = 1.20; 95% CI: 0.33, 2.07), followed by semi-immersive virtual reality (SUCRA = 57.80%; SMD = 0.75; 95% CI: −0.35, 1.86) and nonimmersive virtual reality (SUCRA = 50.90%; SMD = 0.61; 95% CI: −0.15, 1.37). Compared with nonimmersive virtual reality (SUCRA = 50.50%; SMD = 0.42; 95% CI: −0.03, 0.87) and semi-immersive virtual reality (SUCRA = 49.30%; SMD = 0.43; 95% CI: −0.21, 1.07), immersive virtual reality (SUCRA = 95.60%; SMD = 1.11; 95% CI: 0.40, 1.82) was the most effective intervention for hand dexterity. With respect to independence in daily life, immersive virtual reality was the most effective technology (SUCRA = 94.90%; SMD = 1.32; 95% CI: 0.46, 2.18), followed by nonimmersive virtual reality (SUCRA = 43.10%; SMD = 0.28; 95% CI: −0.91, 1.47) and semi-immersive virtual reality (SUCRA = 39.50%; SMD = 0.23; 95% CI: −1.00, 1.46).

**Conclusion:**

Immersive virtual reality is the most effective treatment method for improving upper limb motor function, enhancing hand dexterity, and enhancing independence in the daily life of stroke patients. In the future, clinicians should consider prioritizing immersive virtual reality as a primary modality for stroke rehabilitation exercises.

**Systematic review registration:**

https://www.crd.york.ac.uk/PROSPERO/view/CRD420251114167, Identifier: CRD420251114167.

## 1 Introduction

Stroke is the second leading cause of both disability and death worldwide, with nearly 14 million new cases each year, creating an immense burden on patients, families, and society (1, 2). Upper limb motor function impairment is a common sequelae after stroke, and approximately 80% of stroke survivors develop these sequelae (3). Impaired upper limb motor function and hand dexterity substantially impair independence in activities of daily living and significantly reduce quality of life (4). In addition, with the worsening of these impairments, patients increase their reliance on caregivers, which contributes to psychological distress and social isolation (5). According to recent health statistics, stroke remains a major global health burden and accounts for 5.6% of the total disability-adjusted life years (DALYs) worldwide (6). Therefore, providing essential support to stroke patients is vital for enhancing their upper limb motor function, hand dexterity, and independence in daily life.

Currently, routine rehabilitation approaches are labor intensive and resource demanding (7). In this case, virtual reality (VR) has emerged as a promising therapeutic modality to facilitate motor recovery through neuroplasticity, which is unachievable by routine rehabilitation (8). Compared with routine rehabilitation, VR offers numerous benefits for stroke rehabilitation, which facilitates functional recovery in stroke survivors by delivering high-intensity, repetitive, task-specific training (9, 10). Furthermore, VR accurately simulates complex real-world environments—such as kitchens and shopping malls—thereby facilitating therapeutic activities that would be unsafe or impractical to perform in physical settings (11). For stroke survivors, these VR-based interventions are often perceived as engaging and motivating games, which may significantly improve adherence to rehabilitation regimens (12). Moreover, VR actively engages patients’ senses, particularly vision and hearing. This multisensory stimulation enhances communication within the cortical networks of the brain, promoting neural reorganization and supporting functional recovery (13).

VR systems are classified as immersive virtual reality (IVR; wearing a head-mounted display that completely blocks the sensation of the real world), semi-immersive virtual reality (SIVR; using a large-screen display or headphones for partial immersion), and nonimmersive virtual reality (NIVR; using computer monitors or standard television screens that allow simultaneous existence of real and virtual environments) on the basis of their level of immersion (14). Previous research has demonstrated that different levels of immersion VR improve upper limb motor function, hand dexterity, and independence in daily life among stroke survivors. However, it remains unclear which level of immersion is most effective.

Network meta-analysis allows for the simultaneous comparison and ranking of multiple interventions by integrating direct and indirect evidence, thereby enhancing the precision and reliability of the findings. The findings derived from this approach can support health care professionals in making evidence-based decisions. Thus, the aim of the present study is to perform NMA of different levels of immersion VR for stroke patients to identify which VR has the best effects and to provide evidence for clinical recommendations.

## 2 Methods

The NMA followed the Preferred Reporting Items for Systematic Reviews and Meta-Analyses extension for Network Meta-Analysis (PRISMA-NMA) checklist (15). The protocol was registered in PROSPERO (CRD420251114167).

### 2.1 Search strategy

We systematically searched PubMed, Embase, the Cochrane Library, CINAHL, and Web of Science from database inception to July 2025. Potentially eligible studies were identified for inclusion by manually checking the reference lists of previous pertinent studies and reviews. The search approach combined both subject terms and free terms, utilizing the ‘OR’ and ‘AND’ Boolean operators. The search strategies for each database are provided in Supplementary material S1.

### 2.2 Eligibility criteria

Studies were eligible for the NMA if they met all the following criteria:

(1) Patient characteristics: Adults who had experienced their first ischemic or hemorrhagic stroke and were diagnosed according to clear or internationally recognized diagnostic criteria, without restrictions on race or sex.
(2) Intervention: VR-based rehabilitation, including immersive VR, semi-immersive VR, and nonimmersive VR.
(3) Control (CT): CT, including conventional rehabilitation or VR with a different level of immersion, was compared to the intervention group.
(4) Outcome: Studies in which the effects of VR interventions on stroke survivors were evaluated via standardized assessment tools were included. The outcomes of interest were upper extremity function, hand dexterity, and independence in daily life in response to the intervention.
(5) Study type: RCTs.

Studies were excluded if they met the following criteria:

(1) Studies in which VR was combined with other forms of therapy, such as the combination of VR with robotics or the combination of VR with transcranial electrical stimulation;
(2) Studies that lacked research on the clear duration of individual interventions;
(3) Studies with unclear outcome indicators that could not be accurately extracted or had missing data;
(4) The control group consisted of nonstroke survivors;
(5) Articles that were abstracts, reviews, case reports, or research protocols.

Among repeatedly published studies, the study that provided the most comprehensive data was selected for inclusion.

### 2.3 Study selection

Two reviewers independently screened and reviewed the titles and abstracts of the studies to identify potentially eligible studies and obtained full texts for final inclusion. Disagreements were resolved by discussion with a systematic review team. Study selection was conducted using the review management software Endnote 21.

### 2.4 Data extraction

Two independent reviewers evaluated studies for inclusion through a two-step process. The titles and abstracts were initially screened, followed by a comprehensive full-text assessment based on predetermined inclusion and exclusion criteria to determine final eligibility. Any disagreements were resolved through consultation with a third reviewer or by reference to the original study. To evaluate the effects of VR, data extraction was conducted using a standardized form to collect the following essential information: first author; publication year; study country; intervention and comparison group content and dosage; sample size; and age. Furthermore, elements critical for risk of bias assessment were recorded. In studies featuring multiple groups, only the CT group with a matched treatment dosage matching the VR group was included for comparison.

### 2.5 Quality assessment

Two researchers used the Cochrane Collaboration risk of bias tool (RoB2) (16) to conduct risk assessments involving the following five aspects: selection bias resulting from the randomization process; performance bias resulting from variations from planned interventions; detection bias resulting from missing outcome data; attrition bias resulting from the measurement of the outcome; and overall bias (17). The risk of bias was classified as low, high, or unclear, as described in the Cochrane Handbook.

### 2.6 Statistical analysis

First, we used Review Manager 5.4 for pairwise meta-analyses of all the direct comparisons. For the continuous outcomes, we extracted and calculated the mean and standard deviation of the postintervention data to measure the effect size. Specifically, standardized mean differences (SMD) with 95% confidence intervals (95% CI) were used to express the effect size because not all of the studies utilized the same scale for their specific assessments. The effect size was rated as small (0.2), moderate (0.5), or large (0.8). Heterogeneity was evaluated via the I^2^ test. I^2^ values of 0, 25, 50 and 75% corresponded to no, low, medium and high heterogeneity, respectively. A fixed-effects model was used to calculate the pooled effect sizes if the data were not significantly heterogeneous. Otherwise, a random effects model was used.

We performed a network meta-analysis using STATA 14.2 to synthesize direct and indirect evidence. Direct and indirect evidence from any pair of interventions was combined to generate pooled effect sizes expressed as SMDs with 95% CIs. Global consistency was assessed via inconsistency models, and NMA was performed based on the consistency model to comprehensively compare the efficacy of various virtual reality interventions. The node-splitting method was used to locally test the consistency assumption (18–20). This method involved assessing inconsistency between direct and indirect evidence for specific treatment comparisons within the network. The surface under the cumulative grading curve (SUCRA) was used to separately rank the different virtual reality. A larger SUCRA value corresponded to a higher ranking. A *p* value <0.05 indicated statistical significance. Furthermore, visual inspection of the comparison-adjusted funnel plots was used to assess publication bias.

To verify result stability, we performed sensitivity analyses by leave-one-out exclusion of individual studies and recalculating summary effects. Variations in pooled values and significance were used to assess single-study influence.

## 3 Results

A schematic diagram for the selection of the included studies is shown in Figure 1. In total, 6,803 articles were identified from the establishment of the database to July 2025. Finally, 29 randomized controlled trials, involving 1,524 participants, met the eligibility criteria and were included. The characteristics of the included studies are summarized in Supplementary material S2.

**Figure 1:**
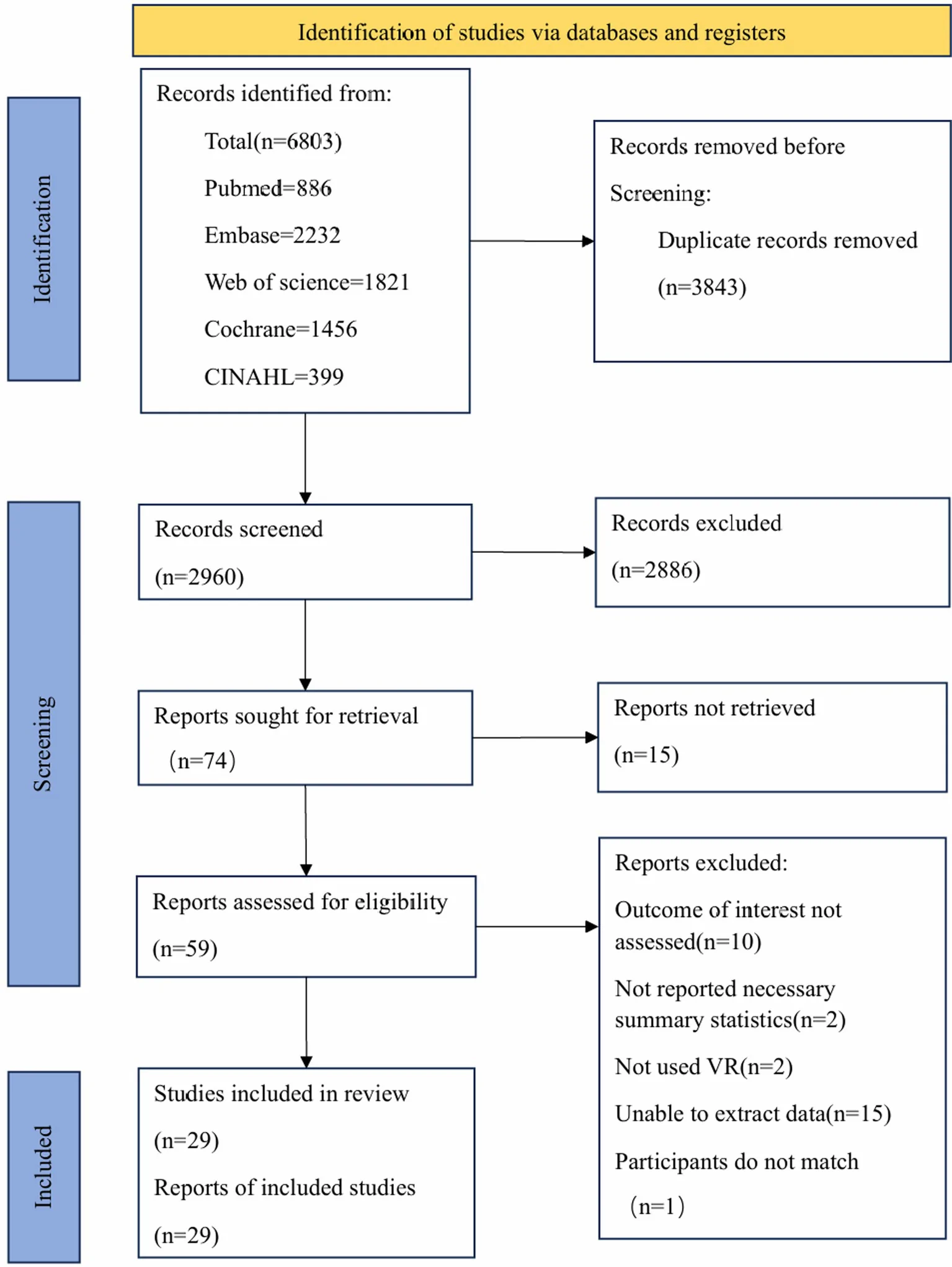
Flow diagram of the search process and selection of studies.

### 3.1 Baseline characteristics and quality of the included studies

Among the included studies 10 studies (9, 21–29) compared IVR with routine rehabilitation, 6 studies (30–35) compared SIVR with routine rehabilitation, and 13 studies (36–48) compared NIVR with routine rehabilitation. Across the included studies, the participant numbers ranged from 12 to 376. Interventions spanned 2–12 weeks and were delivered weekly at a frequency of 2–5 sessions, with session durations varying between 30 min and 3.5 h. Upper limb motor function in stroke survivors was assessed using the Fugl-Meyer Assessment of Upper Extremity (FMA-UE) scale. Hand dexterity was evaluated through the Jebsen-Taylor Hand Function Test (JTT), Box and Block Test (BBT), and Action Research Arm Test (ARAT). Independence of daily life was measured via the Functional Independence Measure (FIM), Barthel Index (BI), and Modified Barthel Index (MBI). For all the scales, higher scores denote superior functional outcomes.

Studies used a variety of VR devices. Several clinical rehabilitation studies adopted standalone VR head-mounted displays, Pico G2 (24), Oculus Quest 2 VR (25), Oculus Rift VR (22), HTC VIVE (23, 26), and 1 study failed to specify device types (29). Most studies employed motion capture devices, Xbox Kinect (27, 30, 31, 33, 34, 38, 40, 42), and Wii (32, 35, 39, 40). Some studies adopted special customized interactive hardware for upper limb rehabilitation, RAPAEL Smart Glove (37, 45, 47), interactive upper-extremity exoskeleton (44), custom somatosensory game controllers (36), and custom scene-sensing interactive hardware (21).

### 3.2 Risk of bias

In general, the RCTs that were included in the present NMA demonstrated an acceptable and relatively low risk of bias. Almost all of the included trials reported adequate random sequence generation; however, most of the studies did not provide sufficient details on allocation concealment, which may have resulted in selection bias. Additionally, the majority of the studies exhibited a high risk of bias in terms of the blinding of participants and personnel, thereby indicating that these aspects were not properly addressed in the study design or implementation. However, the blinding of outcome assessments, incomplete outcome data, selective reporting, and other bias domains generally resulted in a low risk of bias. The detailed risk of bias assessment for each included study is presented in Supplementary Figures S3a,b.

### 3.3 Traditional pairwise meta-analysis

#### 3.3.1 Primary outcome: upper limb motor function

Pairwise meta-analysis (Supplementary Figures S4a,b) showed that compared with the control group, different virtual reality interventions significantly improved the upper limb function of stroke patients: IVR (SMD 1.12, 95% CI 0.43 to 1.80), SIVR (SMD 0.77, 95% CI 0.07 to 1.48), and NIVR (SMD 0.57, 95% CI 0.19 to 0.94). Furthermore, we also conducted subgroup analyses for different types of strokes: chronic stroke (SMD 0.68, 95% CI 0.15 to 1.21), subacute stroke (SMD 0.63, 95% CI 0.24 to 1.03).

#### 3.3.2 Secondary outcome: hand dexterity

Pairwise meta-analysis (Supplementary Figures S4c,d) showed that compared with the control group, different virtual reality interventions significantly improved the hand dexterity of stroke patients: IVR (SMD 1.11, 95% CI 0.37 to 1.86), and SIVR (SMD 0.48, 95% CI 0.20 to 0.77). Furthermore, we also conducted subgroup analyses for different types of strokes: chronic stroke (SMD 0.51, 95% CI 0.24 to 0.79).

#### 3.3.3 Secondary outcome: independence in daily life

Pairwise meta-analysis (Supplementary Figures S4e,f) showed that compared with the control group, only IVR (SMD 1.32, 95% CI 0.42 to 2.23) interventions significantly improved the independence in daily life of stroke patients. Furthermore, we also conducted subgroup analyses for different types of strokes: chronic stroke (SMD 0.83, 95% CI 0.06 to 1.59).

### 3.4 Network meta-analysis

#### 3.4.1 Primary outcome: upper limb motor function

A total of 29 randomized controlled trials (RCTs), encompassing 1,524 participants, evaluated the effects of VR at three different immersion levels on upper limb motor functional recovery after stroke. The global inconsistency test indicated no significant inconsistency across the entire network (*p* = 0.1139). All comparisons in the local inconsistency tests yielded *p* > 0.05, indicating that there was no significant inconsistency at the local level. The detailed results are provided in Supplementary Figures S5a,b. The network diagram is shown in Figure 2, in which each intervention is represented as a node. The node size is proportional to the number of participants assigned to the respective intervention. The lines connecting nodes represent direct comparisons, and the line width is proportional to the number of trials comparing the respective intervention pairs.

**Figure 2:**
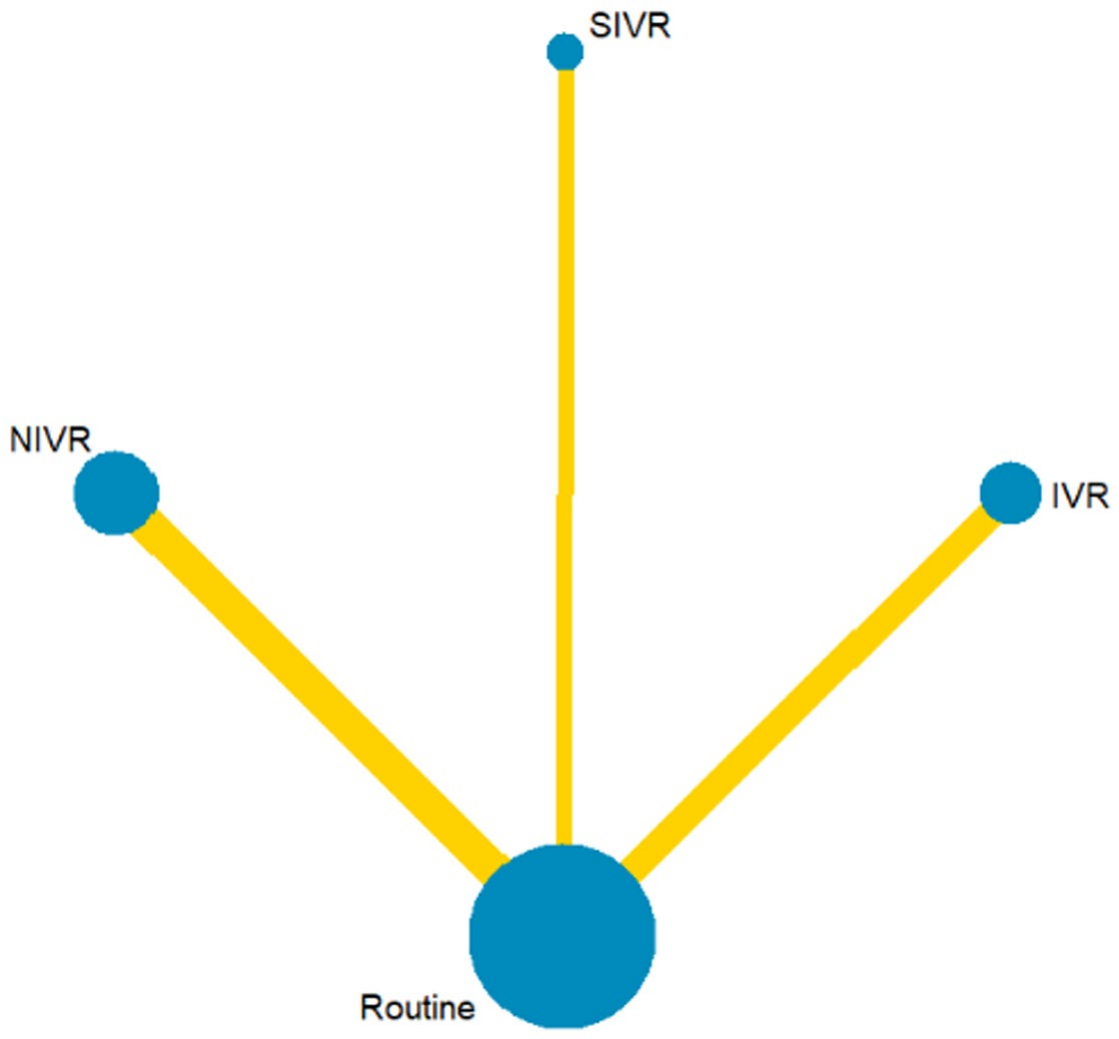
Network map for the comparison of the effects of 3 different immersion levels on upper limb functional recovery after stroke.

The relative effects of the different interventions are presented in Figure 3. Compared with routine rehabilitation, IVR was superior (SMD = 1.20, 95% CI [0.33 to 2.07]). There was no statistically significant difference observed between the other comparisons.

**Figure 3:**
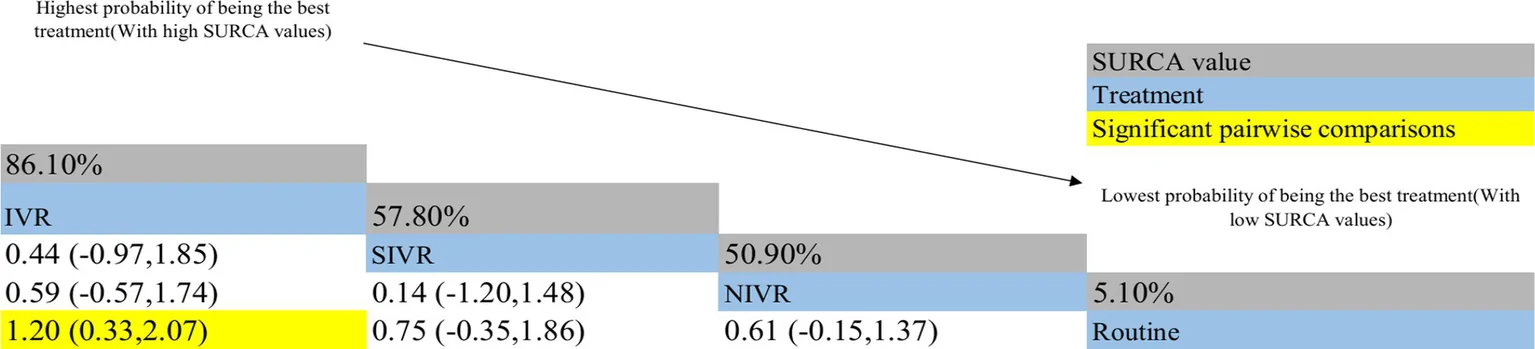
Relative effect sizes of posttreatment efficacy according to the network meta-analysis. The treatments were ranked according to their probability of being the best treatment. The numbers in gray boxes are surface under the cumulative ranking curve (SUCRA) values and their confidence intervals (CI), representing the treatment rankings. Significant pairwise comparisons are highlighted in dark yellow bold font. For posttreatment efficacy, standard mean differences (SMDs) greater than 0 favor the column-defining treatment, and SMDs lower than 0 favor the row-defining treatment. IVR, fully immersive virtual reality; SIVR, semi-immersive virtual reality; NIVR, nonimmersive reality virtual; Routine, routine rehabilitation.

The rank probabilities of the efficacies of the different interventions are shown in Figure 3. IVR was the most likely to be ranked first (86.10%), followed by SIVR (57.80%), NIVR (50.09%) and routine rehabilitation (5.10%). The SUCRA details are presented in Supplementary Figure S5c.

#### 3.4.2 Secondary outcome: hand dexterity

A total of 20 randomized controlled trials (RCTs), encompassing 777 participants, evaluated the effects of VR at three different immersion levels on hand dexterity recovery after stroke. The global inconsistency test indicated no significant inconsistency across the entire network (*p* = 0.0942). All comparisons in the local inconsistency tests yielded *p* > 0.05, indicating that there was no significant inconsistency at the local level. The detailed results are provided in Supplementary Figures S6a,b. The network diagram is shown in Figure 4.

**Figure 4:**
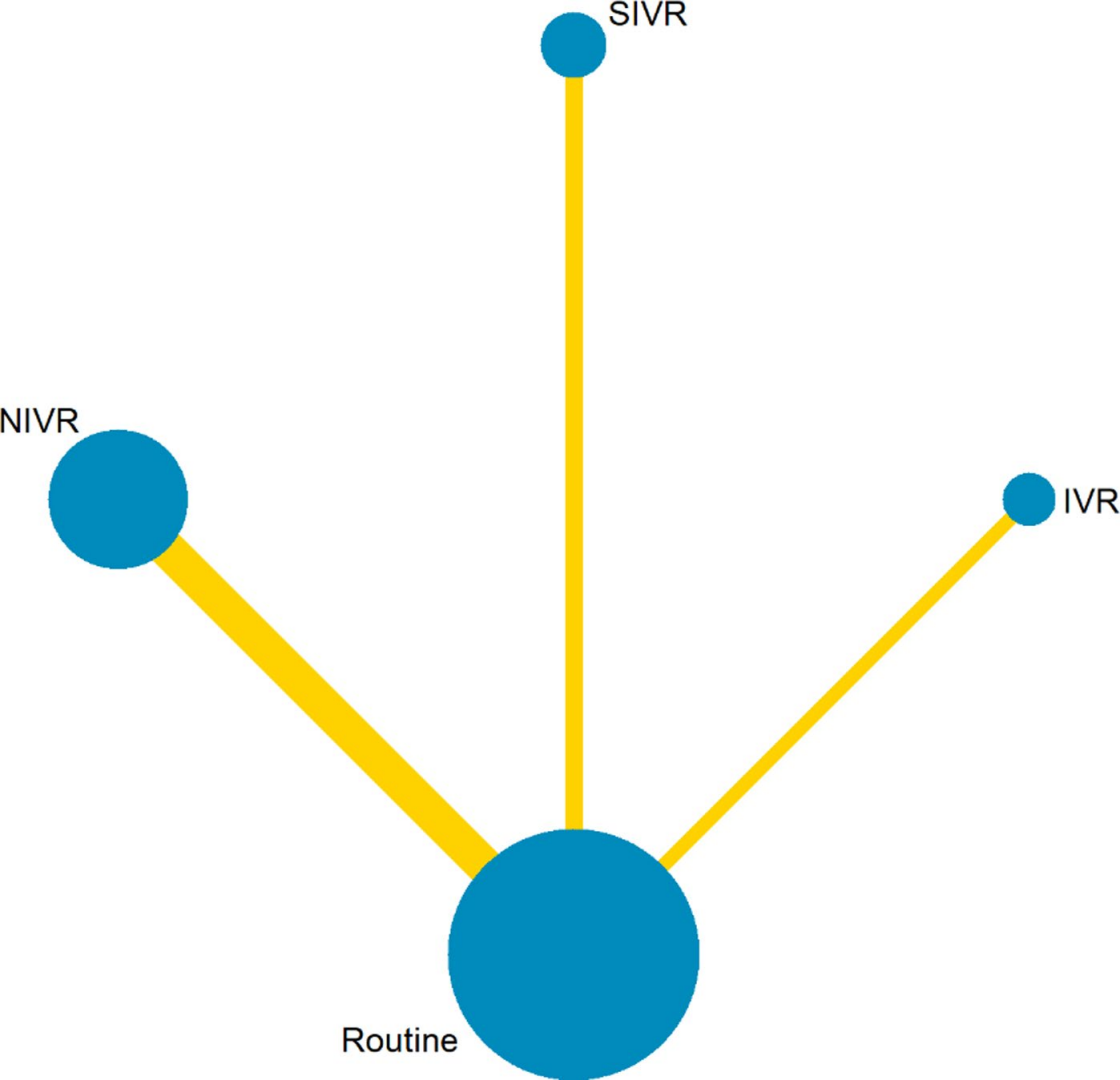
Network map for the comparison of 3 different immersion levels on hand dexterity after stroke.

The relative effects of the different interventions are presented in Figure 5. IVR was more effective than routine rehabilitation (SMD = 1.11, 95% CI [0.40 to 1.82]). There was no statistically significant difference observed between the other comparisons.

**Figure 5:**
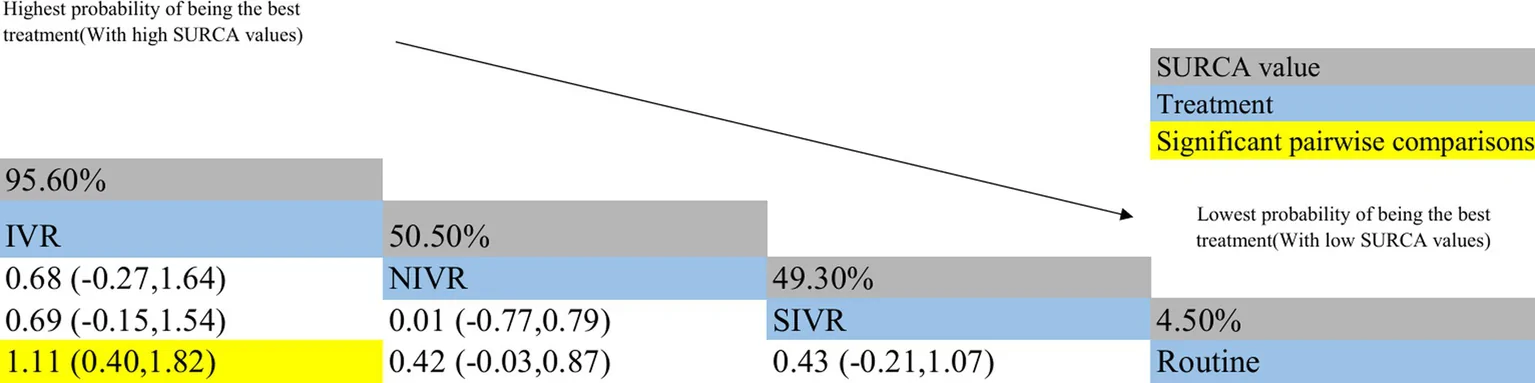
Relative effect sizes of posttreatment efficacy according to the network meta-analysis. The treatments were ranked according to their probability of being the best treatment. The numbers in gray boxes are surface under the cumulative ranking curve (SUCRA) values and their confidence intervals (CI), representing the treatment rankings. Significant pairwise comparisons are highlighted in dark yellow bold font. For posttreatment efficacy, standard mean differences (SMDs) greater than 0 favor the column-defining treatment, and SMDs lower than 0 favor the row-defining treatment. IVR, fully immersive virtual reality; SIVR, semi-immersive virtual reality; NIVR, nonimmersive reality virtual; Routine, routine rehabilitation.

The rank probabilities of the efficacies of different interventions are shown in Figure 5. IVR was the most likely to be ranked first (95.60%), followed by NIVR (50.50%), SIVR (49.30%) and routine rehabilitation (4.50%). The SUCRA details are presented in Supplementary Figure S6c.

#### 3.4.3 Secondary outcome: independence in daily life

A total of 12 randomized controlled trials (RCTs), encompassing 919 participants, evaluated the effects of VR at three different immersion levels on independence in daily life recovery after stroke. The global inconsistency test indicated no significant inconsistency across the entire network (*p* = 0.6621). All comparisons in the local inconsistency tests yielded *p* > 0.05, indicating that there was no significant inconsistency at the local level. The detailed results are provided in Supplementary Figures S7a,b. The network diagram is shown in Figure 6.

**Figure 6:**
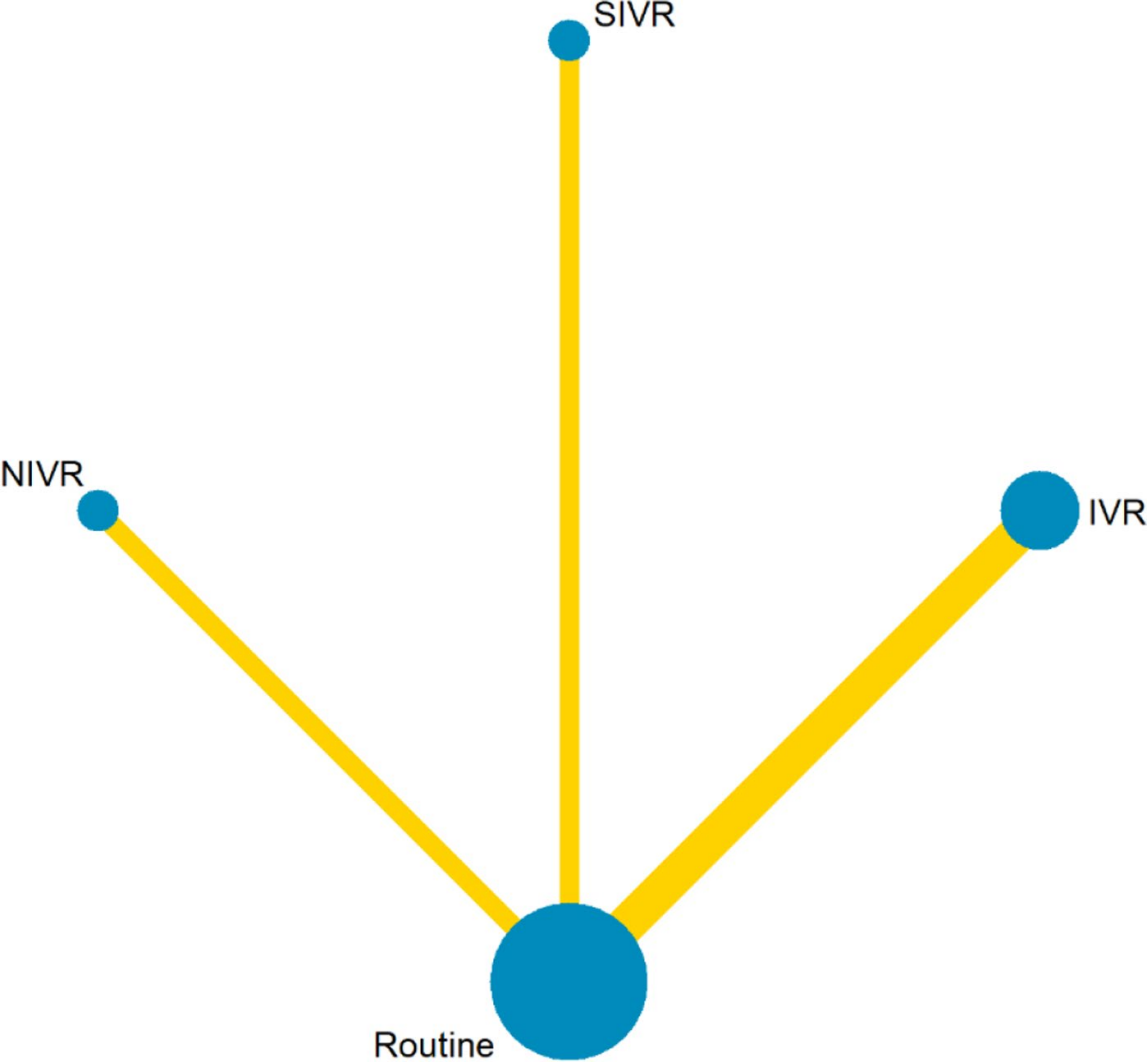
Network map for the comparison of the effects of 3 different immersion levels on independence in daily life after stroke.

The relative effects of the different interventions are presented in Figure 7. IVR was superior to routine rehabilitation (SMD = 1.32, 95% CI [0.46 to 2.18]). There was no statistically significant difference observed between the other comparisons.

**Figure 7:**
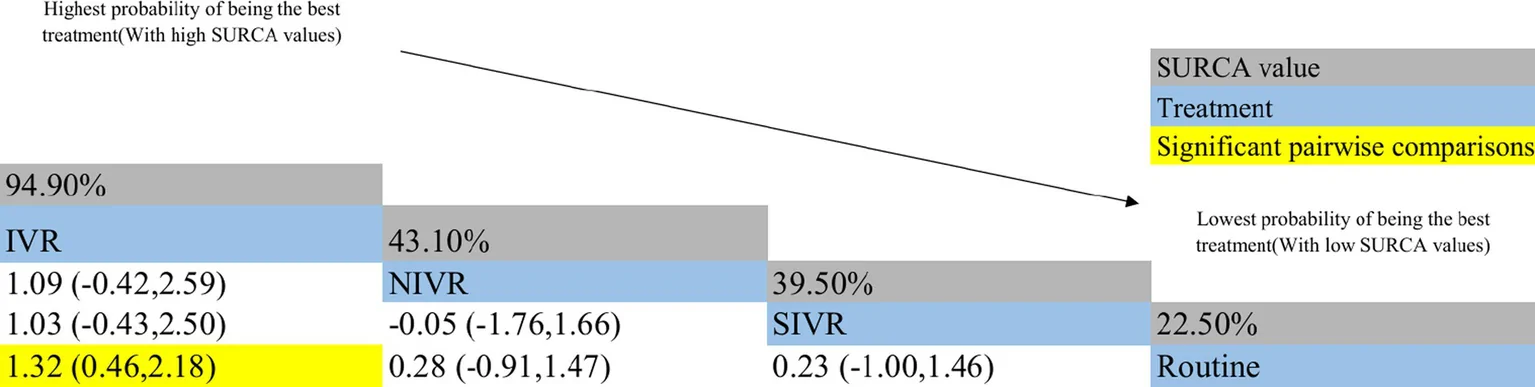
Relative effect sizes of posttreatment efficacy according to the network meta-analysis. The treatments were ranked according to their probability of being the best treatment. The numbers in gray boxes are surface under the cumulative ranking curve (SUCRA) values and their confidence intervals (CI), representing the treatment rankings. Significant pairwise comparisons are highlighted in dark yellow bold font. For posttreatment efficacy, standard mean differences (SMDs) greater than 0 favor the column-defining treatment, and SMDs lower than 0 favor the row-defining treatment. IVR, fully immersive virtual reality; SIVR, semi-immersive virtual reality; NIVR, nonimmersive reality virtual; Routine, routine rehabilitation.

The rank probabilities of the efficacies of different interventions are shown in Figure 7. IVR was the most likely to be ranked first (94.60%), followed by NIVR (43.10%), SIVR (39.50%) and routine rehabilitation (22.50%). The SUCRA details are presented in Supplementary Figure S7c.

### 3.5 Publication bias

We assessed the potential for publication bias and small-study effects within the network by visually inspecting the comparison-adjusted funnel plots for all pairwise comparisons contributing to the network meta-analysis. No evidence of publication bias was detected, as assessed by the symmetry of the comparison-adjusted funnel plots. The comparison-adjusted funnel plots are shown in Supplementary Figures S8a–c.

### 3.6 Sensitivity analysis

Sensitivity analysis via leave-one-out sequential study exclusion was performed to assess the robustness of pooled estimates. After recalculating pooled effect sizes upon omitting one individual study at a time, all updated point estimates fluctuated marginally around the original overall pooled effect. No reversal of statistical significance was observed across all 95% confidence intervals, and no single study could drastically alter the overall statistical conclusion. Collectively, the pooled Meta-analysis results exhibited low susceptibility to perturbations from individual included studies, supporting favorable robustness and reliability of the conclusions. The Sensitivity analysis is shown in Supplementary material S9.

## 4 Discussion

This NMA evaluated the effects of VR interventions at varying immersion levels on upper limb motor function, hand dexterity, and independence in the daily life of stroke survivors. IVR consistently yielded the most substantial improvements across all outcome measures—upper limb motor function, hand dexterity, and independence in daily life—poststroke. These findings may inform clinical decision-making for health care professionals who are developing targeted VR rehabilitation strategies for stroke survivors.

Compared with routine rehabilitation, the NMA revealed that different levels of immersion virtual reality intervention enhanced upper limb motor function, improved hand dexterity, and increased independence in daily life among stroke survivors; these findings are broadly consistent with those of previous studies (9, 24, 25, 49, 50). In addition, the NMA suggested that IVR may have the best efficacy for improving upper limb motor function. Compared with SIVR and NIVR, IVR presents a virtual environment that is more realistic, more personalized and more intuitive and stimulates more senses, thus increasing the transferability of the skills learned in the virtual environment into real life. For example, higher levels of immersion in virtual reality environments provide more or stronger visual and auditory stimuli for patients (14, 22, 51). In addition, the mechanism underlying the improvement in upper limb motor function may be related to neuroplasticity (26). IVR provides the core elements of neuroplasticity (feedback, repetition, intensity, task-specific training, altering task difficulty, increasing motivation and engagement of the user, and increasing enjoyment of intensive task-relevant training) (52). Huang et al. (23) used brain-derived neurotrophic factor (BDNF) to measure neuroplasticity, and they reported that BDNF expression is significantly improved in the VR group after intervention, indicating that IVR stimulates neuroplasticity and promotes improvements in functional movement of the patient’s upper limb. Furthermore, functional magnetic resonance imaging (fMRI) neuroimaging corroborates these neuroplastic changes. The fMRI results of a study showed distinct neural remodeling patterns induced by IVR rehabilitation in subacute stroke survivors. IVR training significantly elevated activation within the ipsilesional primary motor cortex (M1), premotor cortex and dorsolateral prefrontal cortex, and strengthened interregional functional connectivity of sensorimotor networks. Notably, fMRI-measured connectivity enhancements positively correlated with upper-extremity FMA-UE recovery (9). Consistently, a systematic review pooling multiple fMRI trials further validated such plastic brain shifts. The review identified post-intervention neural reorganization across ipsilesional and contralesional primary motor, sensorimotor, and supplementary motor areas, and these cortical remodeling alterations were frequently accompanied by measurable functional recovery (53).

The present hand dexterity analyses suggested that IVR may be the most effective treatment. Both IVR and SIVR were also more effective than the routine treatment. First, IVR can be effectively integrated with mirror therapy. This approach employs VR technology integrated with head-mounted displays to generate a high-fidelity mirror image of the patient’s hand, utilizing actual visual input rather than computer-generated imagery. Providing an accurate visual representation of the affected limb facilitates a more realistic perception of movement execution, thereby enhancing the recovery of hand motor dysfunction and coordination abilities in stroke survivors (22, 24). In addition, mirror therapy reduces interhemispheric activation asymmetry, stimulates the primary motor cortex in both the ipsilesional and contralesional hemispheres, broadly activates the mirror neuron system, and induces partial motor pathway recruitment in the stroke-affected hemisphere, thereby facilitating functional brain reorganization (54). Second, electroencephalography (EEG) evidence further supports the superior neuromodulatory effects of IVR. Compared with SIVR and NIVR, which only triggers slight mu attenuation confined to the intact hemisphere, IVR mirror feedback yielded prominent mu suppression across ipsilesional primary motor cortex and parietal regions among chronic stroke patients (55). Moreover, IVR intervention boosted motor-control-related beta power and lowered abnormal theta power indicative of cortical impairment; these two EEG markers independently predicted long-term improvements in hand dexterity (56).

The present findings revealed that IVR may be the best intervention for the outcome of independence in daily life. First, IVR provides highly realistic simulations of essential daily living activities—such as shopping, operating light switches, and cooking—serving as task-specific training that directly targets the core skills needed to regain independent living (9, 29). Second, IVR provides a relatively safe training environment for stroke survivors, enabling repetitive practice of essential activities of daily living without the risk of physical harm during task execution errors. This critical safety advantage cannot be replicated in SIVR, NIVR or routine rehabilitation approaches (57). Furthermore, in VR-based rehabilitation systems, the integration of gamification elements, reward systems, and personalized training protocols significantly enhances patient motivation and treatment adherence. This increased engagement promotes consistent participation in daily activity training, thereby contributing to improved independence in daily life among stroke survivors (58). The superior functional recovery induced by IVR can also be substantiated by neuroimaging biomarkers from functional near-infrared spectroscopy (fNIRS) measurements. The fNIRS data from the relevant study show IVR rehabilitation induced larger increases in blood oxygenation level-dependent (BOLD) signals in ipsilesional M1 than SIVR and NIVR, reflecting stronger neuronal firing during repetitive virtual limb training (59). Additional fNIRS evidence further supports the neural advantages of IVR for ADL rehabilitation. Randomized controlled trials utilizing fNIRS to monitor cortical hemodynamics have confirmed prominent task-related cerebral activation during digital functional training, with measurable cortical hemodynamic changes closely linked to improvements in motor and daily living function (37, 60).

From a clinical perspective, IVR may be considered a promising rehabilitation strategy, particularly in healthcare settings with sufficient technological resources and professional support. The immersive environment, enhanced sensory feedback, and task-specific training provided by IVR may facilitate patient engagement and functional recovery. However, implementation should be guided by clinical context, accessibility, and economic considerations. Compared with SIVR and NIVR, IVR requires more advanced hardware, maintenance, and technical expertise, which may limit its widespread adoption. Therefore, future research should incorporate economic evaluations alongside clinical outcomes to determine the feasibility of large-scale implementation. Moreover, the findings should be interpreted cautiously because of substantial heterogeneity among studies, including differences in participant characteristics, intervention duration, training intensity, and VR systems. Although no significant publication bias was identified, small study effects cannot be completely excluded due to the limited number of trials in some comparisons. Future multicenter randomized controlled trials with standardized intervention protocols and long-term follow-up are needed to confirm the comparative effectiveness of different VR immersion levels.

The present review had several limitations. First, the number of VR interventions included in the present study was limited, which may limit the statistical power for comparisons. Second, as no studies directly compared VR delivery formats with other formats, the position of VR delivery formats in the rank was established by circumstantial evidence. Future studies should directly compare the effects between different VR delivery formats to address the relevant knowledge gaps. Finally, the effectiveness data were analyzed from baseline to postintervention, and the conclusions were focused on short-term effects rather than long-term effects. Future studies should investigate outcomes associated with different VR delivery modalities and include follow-up assessments to evaluate long-term efficacy.

## 5 Conclusion

In summary, the present NMA evaluated the effects of different levels of immersion in virtual reality interventions on upper limb motor function, hand dexterity, and independence in the daily life of stroke survivors. The present results indicated that IVR is the most effective delivery format for improving these outcomes in stroke survivors, suggesting that IVR may be considered a promising rehabilitation option, particularly when appropriate resources and technical support are available. The present study provides a valuable evidence base for clinicians to inform their therapeutic strategies. However, critical limitations were identified, including the absence of direct comparisons between distinct immersion tiers. Consequently, future research should explore the effects of virtual reality interventions at different immersion levels on stroke rehabilitation outcomes.

## Data Availability

Publicly available datasets were analyzed in this study. Due to the nature of this research, participants of this study did not agree for their data to be shared publicly, so supporting data is not available.
