# Supplementary material for: Comparative efficacy of virtual reality interventions with varying immersion levels on upper limb motor function, hand dexterity, and independence in the daily life of stroke survivors: a systematic review and network meta-analysis
Source: Front Neurol. 2026 Sep 23;17:1797313. doi: 10.3389/fneur.2026.1797313 (PMC13642267; doi:10.3389/fneur.2026.1797313)

Supplementary material

S1. Search Strategy
S2. Characteristics of the studies included
S3. Risk of bias in the included studies
S4. The results of traditional pairwise meta-analysis
S5. The results of network meta-analysis related to upper limb motor function
S6. The results of network meta-analysis related to hand dexterity
S7. The results of network meta-analysis related to independence in daily life
S8. Funnel plot of all studies
S9. Sensitivity Analysis Using Leave-one-out Method

S1. Search Strategy

PubMed：

#1("Stroke"[Mesh]) OR ((((((Stroke*[Title/Abstract]) OR (Cerebrovascular Accident*[Title/Abstract])) OR (CVA[Title/Abstract])) OR (Cerebrovascular Apoplexy[Title/Abstract])) OR (Brain Vascular Accident*[Title/Abstract])) OR (CVAS[Title/Abstract]))
#2("Virtual Reality"[Mesh]) OR (((((Virtual Reality[Title/Abstract]) OR (virtual environment[Title/Abstract])) OR (VR[Title/Abstract])) OR (kinect[Title/Abstract])) OR (wii gam*[Title/Abstract]))
#3("Exergaming"[Mesh]) OR (((Active-Video Gam*[Title/Abstract]) OR (Active Video Gam*[Title/Abstract])) OR (Exergam*[Title/Abstract]))
#4("Wearable Electronic Devices"[Mesh]) OR (((Wearable Device*[Title/Abstract]) OR (Wearable Technolog*[Title/Abstract])) OR (Electronic Skin[Title/Abstract]))
#5("Video Games"[Mesh]) OR (((Video Gam*[Title/Abstract]) OR (Computer Gam*[Title/Abstract])) OR (interactive gam*[Title/Abstract]))
#6("Mirror Movement Therapy"[Mesh]) OR ((Mirror Therap*[Title/Abstract]) OR (Mirror Movement Therap*[Title/Abstract]))
#7 #2 OR #3 OR #4 OR #5 OR #6
#8 ("Randomized Controlled Trial" [Publication Type]) OR ((((Randomized controlled trial[Title/Abstract]) OR (RCT[Title/Abstract])) OR (controlled clinical trial[Title/Abstract])) OR (random*[Title/Abstract]))
#9 #1 AND #7 AND #8

Embase：

#1 **stroke***:ab,ti OR **'cerebrovascular accident*'**:ab,ti OR **cva**:ab,ti OR **'cerebrovascular apoplexy'**:ab,ti OR **'brain vascular accident*'**:ab,ti OR **cvas**:ab,ti
#2 **'virtual reality'**:ab,ti OR **'virtual environment'**:ab,ti OR **vr**:ab,ti OR **kinect**:ab,ti OR **'wii gam*'**:ab,ti OR **exergam***:ab,ti OR **'active-video gam*'**:ab,ti OR **'active video gam*'**:ab,ti OR **'wearable electronic device*'**:ab,ti OR **'wearable device*'**:ab,ti OR **'wearable technolog*'**:ab,ti OR **'electronic skin'**:ab,ti OR **'video gam*'**:ab,ti OR **'computer gam*'**:ab,ti OR **'interactive gam*'**:ab,ti OR **'mirror therap*'**:ab,ti OR **mt**:ab,ti
#3 'randomized controlled trial':ab,ti OR rct:ab,ti OR 'controlled clinical trial':ab,ti OR random*:ab,ti
#4 #1 AND #2 AMD #3

Cochrane：

#1 (stroke*):ti,ab,kw OR (Cerebrovascular Accident*):ti,ab,kw OR (CVA*):ti,ab,kw OR (Cerebrovascular Apoplexy):ti,ab,kw OR (Brain Vascular Accident*):ti,ab,kw 81901
#2 (virtual reality):ti,ab,kw OR (virtual environment):ti,ab,kw OR (VR):ti,ab,kw OR

(kinect):ti,ab,kw OR (wii gam*):ti,ab,kw 11146
#3 (Wearable Device*):ti,ab,kw OR (Wearable Technolog*):ti,ab,kw OR (Electronic Skin):ti,ab,kw 3232
#4 (interactive gam*):ti,ab,kw OR (Video Gam*):ti,ab,kw OR (Computer Game*):ti,ab,kw 4891
#5 (Mirror Movement Therap*):ti,ab,kw OR (Mirror Therapy):ti,ab,kw 1403
#6 #2 or #3 or #4 or #5 19294
#7 (Randomized Controlled Trial):ti,ab,kw OR (RCT):ti,ab,kw OR (randomized controlled trial):ti,ab,kw OR (controlled clinical trial):ti,ab,kw OR (random*):ti,ab,kw 1459223
#8 #1 and #6 and #7 1483

Web of science:

#1 TS=(Stroke* or Cerebrovascular Accident* or CVA* or Cerebrovascular Apoplexy or Brain Vascular Accident*)
#2 TS=(Virtual Reality or virtual* or vr or Kinect or wii gam* or Wearable Device* or Wearable Technolog* or Exergam* or Video Gam* or Computer Gam* or interactive gam* or Mirror Therap*)
#3 TS=(Randomized controlled trial or RCT or controlled clinical trial or random*)

EBSCO:

#1 stroke* or cerebrovascular accident* or cva or Cerebrovascular Apoplexy or Brain Vascular Accident*
#2 Video Gam* or Computer Gam* or interactive gam* or Wearable Device* or Wearable Technolog* or Exergam* or Virtual Reality or VR or or Kinect or wii gam* or Mirror Therap*
#3 randomized controlled trial* or rct or controlled clinical trial* or random*

S2. Characteristics of the studies included

Table 1 Characteristics of included studies.

author,year	country	type of stroke	type of VR	sample, size(IG/CG)	year(mean±SD, IG/CG)	Intervention	Comparator	Intervention duration
Ain2021	Pakistan	Chronic stroke	SIVR	IG=25<br>CG=25	IG=57.48±10.60<br>CG=57.68±10.43	Based on the regular training, the training was conducted using Xbox Kinect 360	Only undergo conventional physical therapy	5 days a week, 35-40 minutes each time, for a total of 6 weeks
Bai2022	China	-	IVR	IG=25<br>CG=25	IG=54.2±13.7<br>CG=56.9±14.6	Based on traditional treatment methods, one undergoes training using VR technology	Only undergo conventional physical therapy	40-60 minutes per day, 5 days a week, for a total of 10 weeks
Huang2024	China	Subacute stroke	IVR	IG=20<br>CG=20	IG=63.3±14.3<br>CG=65.1±6.1	After the first 30 minutes of conventional rehabilitation, the imVR training was conducted 30 minutes later	Only undergo conventional physical therapy	60 minutes each time, 5 times a week, for a total of 3 weeks
Hsu2022	China	Chronic stroke	IVR	IG=18<br>CG=17	IG=52.9±11.8<br>CG=56.9±13.0	20-minute task-specific training + 30-minute VR-MT training	20-minute task-specific training + 30-minute regular occupational therapy	50 minutes each time, twice a week, for a total of 9 weeks

Huang2022	China	Chronic stroke	IVR	IG=15<br>CG=15	IG=50.80±12.32<br>CG=58.33±11.22	Using HTC VIVE for virtual reality training, the training tasks include aiming and shooting, and some of the tasks require coordination of both hands	Perform upper limb training using conventional equipment such as peg boards and climbing ladders	Every 60 minutes, 2-3 times a week, for a total of 16 times
Johnson2020	Australian	Chronic stroke	SIVR	IG=28<br>CG=30	IG=64.7±13.9<br>CG=59.3±15.6	The virtual reality training was conducted using the Jintronix rehabilitation system. The training content included customized games for the upper limbs (4-6 in total)	Maintain daily activities and treatment plans, and do not accept any additional interventions	Each session lasts about 45 minutes, twice a week, for a total of 8 weeks
Lam2022	China	Subacute stroke	NIVR	IG=47<br>CG=46	IG=65.1±10.02<br>CG=66.0±9.0	3-hour regular rehabilitation (CR) + 30-minute bilateral motor computer game training	3-hour regular rehabilitation (CR) + 30-minute video-guided exercise	Twice a week, for 3.5 hours each time, for a total of 8 weeks
Mekbib2021	China	Subacute stroke	IVR	IG=12<br>CG=11	IG=52.17±13.26<br>CG=61.00±7.69	1 hour of MNVR-Rehab training + 1 hour of regular occupational therapy	2-hour regular occupational therapy	2 hours each time, 4 days a week, for a total of 2 weeks

Shin2022	Korea	Subacute stroke	NIVR	IG=20<br>CG=16	IG=57.00±12.78<br>CG=63.69±8.58	30 minutes of RAPAEL® intelligent glove gamified virtual reality training + 30 minutes of conventional occupational therapy	60-minute regular exercise therapy	Once per hour, 5 days a week, for a total of 4 weeks
Sungbae2024	Korea	Chronic stroke	IVR	IG=15<br>CG=15	IG=51.73±13.63<br>CG=47.13±13.91	60-minute conventional exercise therapy+30 minutes 360-degree immersive virtual reality mirror training	60-minute regular physical therapy	IG:Every 90 minutes, 3 times a week, for a total of 4 weeks<br>CG:Every 60 minutes , 5 times a week, for a total of 4 weeks
Sin2013	Korea	Chronic stroke	SIVR	IG=18<br>CG=17	IG=71.78±9.42<br>CG=75.59±5.55	30 minutes of Xbox Kinect virtual reality training+30 minutes of regular occupational therapy	30-minute regular exercise therapy	IG:60 minutes each time, 3 times a week, for a total of 6 weeks<br>CG:30 minutes each time, 3 times a week, for a total of 6 weeks
Amin2024	Pakistan	Subacute stroke	IVR	IG=26<br>CG=26	IG=51.8±12.9<br>CG=49.8±9.9	1-2 weeks: 24 minutes of VR games every day+ + 24 minutes of regular physical therapy,3-6 weeks: 40 minutes of VR games every day+40 minutes of regular physical therapy	1-2 weeks: 48 minutes of regular physical therapy per day,3-6 weeks: 80 minutes of regular physical therapy per day	4 days a week, for a total of 6 weeks

Elena2021	Spain	Chronic stroke	SIVR	IG=15<br>CG=14	IG=61.5±8.4<br>CG=58.2±7.4	Conventional physical therapy + Nintendo Wii training	Conventional physical therapy	Twice a week, 2 hours each time, for a total of 4 weeks
Sevgi2018	Turkey	Subacute stroke	SIVR	IG=19<br>CG=16	IG=69.42±8.55<br>CG=63.44±15.73	Regular rehabilitation training + additional Xbox Kinect training	Only accept the aforementioned regular rehabilitation training	IG:Regular rehabilitation lasts for 60 minutes each time, while Xbox Kinect training lasts for 30 minutes each time. It is conducted 5 times a week for a total of 4 weeks<br>CG:60 minutes each time, 5 times a week, for a total of 4 weeks
Choi2014	Korea	Subacute stroke	SIVR	IG=10<br>CG=10	IG=64.30±10.3<br>CG=64.70±11.3	Wii game training conducts upper limb movement training through specific games	Routine occupational therapy	30 minutes per session, 5 times a week, for a total of 4 weeks
Patrícia2019	Brazil	Chronic stroke	IVR	IG=16<br>CG=15	IG=76.19±10.09<br>CG=76.20±10.41	Motion Rehab AVE 3D Kinesthetic Game Training, including upper limb and balance-related movement training	Conventional physical therapy	30 minutes per session, twice a week, for a total of 12 weeks
Kim2018	Brazil	Subacute stroke	NIVR	IG=12<br>CG=11	IG=56.7±17.8<br>CG=57.2±15.0	Regular occupational therapy + Additional realistic VR training	Regular occupational therapy + additional virtual reality training	Every 60 minutes, for a total of 10 days

Kiper2018	Italy	Subacute or chronic stroke	IVR	IG=68<br>CG=68	IG=62.5±15.2<br>CG=66.0±12.9	Training in a virtual reality environment combined with conventional rehabilitation	Routine rehabilitation	2 hours per day, 5 days a week, for a total of 4 weeks
Kong2016	Singapore	-	NIVR	IG=35<br>CG=35	IG=58.1±9.1<br>CG=55.8±11.5	Nintendo Wii game training + regular rehabilitation	Only accepts conventional rehabilitation	60 minutes per session, 4 times a week, for a total of 3 weeks
Kottink2014	Netherlands	Chronic stroke	NIVR	IG=8<br>CG=10	IG=65.3±6.5<br>CG=58.4±14.8	"Furball Hunt" Rehabilitation Game Training	Standardized routine reaching exercises	30 minutes per session, 3 times a week, for a total of 6 weeks
Levin2012	Canada	Chronic stroke	NIVR	IG=6<br>CG=6	IG=58.1±14.6<br>CG=59.8±15.1	VR training is mainly based on goal-oriented hand movements, and the difficulty level adjusts according to one's ability	Conventional occupational therapy	45 minutes per session, 3 times a week, for a total of 3 weeks
Norouzi-Gheidari2019	Canada	Subacute or chronic stroke	NIVR	IG=9<br>CG=9	IG=42.2±9.5<br>CG=57.6±10.5	Conventional rehabilitation therapy + Additional Jintronix system training	Only accepts conventional rehabilitation treatment	30 minutes each time, 2-3 times a week, for a total of 4 weeks
Ögün2019	Turkey	-	IVR	IG=33<br>CG=32	IG=61.48±10.92<br>CG=59.75±8.07	3D immersive VR rehabilitation training, featuring 4 targeted games	Conventional upper limb active training, pseudo-VR training	60 minutes per session, 3 times a week, for a total of 6 weeks

Oh2019	Korea	-	NIVR	IG=17<br>CG=14	IG=57.4±12.2<br>CG=52.6±10.7	VR combined with real equipment training, using the designated system and equipment	Conventional occupational therapy	30 minutes per session, 3 times a week, for a total of 6 weeks
Chen2022	China	Subacute stroke	NIVR	IG=18<br>CG=18	not clear	VR training, including tasks such as "boiling eggs", "picking apples" and "archery", each lasting for 15 minutes	Conventional occupational therapy	60 minutes per session, 5 times a week, for a total of 2 weeks
Shin2016	Korea	-	NIVR	IG=24<br>CG=22	IG=57.2±10.3<br>CG=59.8±13.0	Standard Occupational Therapy (OT) + Smart Glove VR Rehabilitation	Standard occupational therapy (OT) + conventional rehabilitation training	60 minutes per session, 5 times a week, for a total of 4 weeks
Turolla2013	Italy	-	NIVR	IG=263<br>CG=113	IG=60.2±14.3<br>CG=65.4±12.5	Conventional upper limb rehabilitation + Virtual environment enhanced feedback training	Conventional upper limb rehabilitation	2 hours per session, 5 times per week, for a total of 4 weeks
Ase2025	Japan	Chronic stroke	NIVR	IG=7<br>CG=7	IG=58.0±6.4<br>CG=51.4±6.7	Wear the RAPAEL Smart Glove for home training	Regular upper limb training at home	30 minutes each time, 5 days a week, for a total of 4 weeks
Piron2009	Italy	-	NIVR	IG=18<br>CG=18	IG=66.0±7.9<br>CG=64.4±7.9	The VRRS.net® system offers remote rehabilitation training	Conventional physical therapy	Once every hour, 5 days a week, for a total of 4 weeks

Note: IVR: immersive virtual reality, SIVR: semi-immersive virtual reality, NIVR: non-immersive virtual reality.

S3. Risk of bias in the included studies

Study ID	D1	D2	D3	D4	D5	Overall
Ain2021	+	+	+	+	?	!
Bai2022	+	+	+	?	?	!
Huang2024	+	+	+	+	?	+
Hsu2022	+	+	+	+	?	!
Huang2022	+	+	+	+	+	+
Johnson2020	+	+	+	+	?	!
Lam2022	+	+	+	+	?	!
Shin2022	+	+	+	+	? (red)	? (red)
Sungbae2024	+	+	+	+	?	!
Sin2013	?	?	+	+	? (red)	? (red)
Amin2024	?	+	+	?	? (red)	? (red)
Elena2021	+	+	+	+	?	!
Sevgi2018	?	+	+	+	?	!
Choi2014	?	+	+	+	?	!
Patrícia2019	?	+	+	?	?	!
Kim2018	+	+	+	+	? (red)	? (red)
Kiper2018	+	+	+	+	?	!
Kong2016	+	? (red)	+	+	? (red)	? (red)
Kottink2014	?	+	+	+	? (red)	? (red)
Levin2012	?	+	+	+	?	!
Norouzi-Gheidari2019	?	? (red)	+	+	? (red)	? (red)
Ögün2019	+	? (red)	+	+	? (red)	? (red)
Oh2019	+	+	+	+	?	+
Chen2022	+	+	+	+	?	!
Shin2016	+	+	+	+	?	!
Turolla2013	? (red)	+	+	+	? (red)	? (red)
Ase2025	?	+	+	+	?	!
Mekbib2021	+	+	+	+	?	+
Piron2009	+	?		+	?	!

\+ Low risk
? Some concerns
? (red) High risk
D1 Randomization process
D2 Deviations from intended interventions
D3 Missing outcome data
D4 Measurement of the outcome
D5 Selection of the reported result

S3a. Risk-of-bias distribution across studies

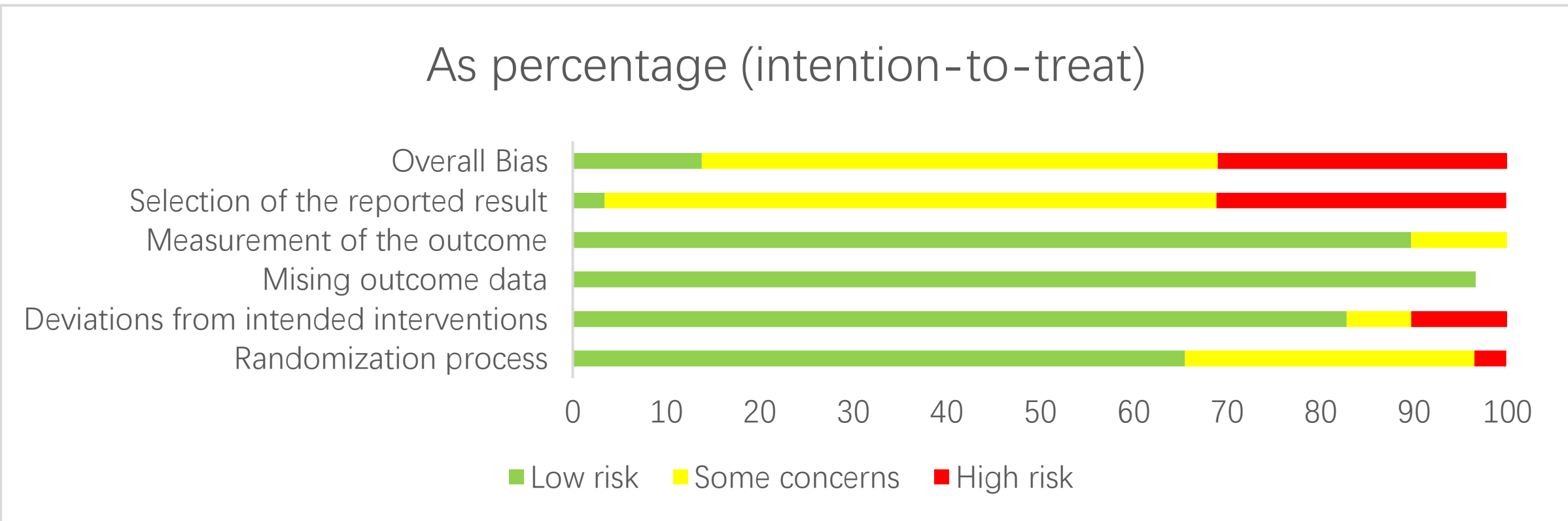

S3b. Risk of bias graph: review authors' judgement about each risk of bias item, presented as percentages across all included studies.

S4. The results of traditional pairwise meta-analysis

Study or Subgroup	Experimental Mean	Experimental SD	Experimental Total	Control Mean	Control SD	Control Total	Weight	Std. Mean Difference IV, Random, 95% CI
**1.2.1 Immersive virtual reality**								
Amin2024	55.92	7.3	26	42.23	7.86	26	3.6%	1.78 [1.13, 2.43]
Bai2022	47.46	0.48	25	41.82	0.79	25	1.7%	8.49 [6.67, 10.31]
Hsu2022	46.1	13.7	18	35.1	18.6	17	3.6%	0.66 [-0.02, 1.34]
Huang2022	52.47	9.11	15	45.53	16.39	15	3.5%	0.51 [-0.22, 1.24]
Huang2024	34.9	18.4	20	24	20.7	20	3.7%	0.55 [-0.09, 1.18]
Kiper2018	47.71	15.74	68	46.29	17.25	68	4.1%	0.09 [-0.25, 0.42]
Mekbib2021	12.25	4.58	12	7.7	2.54	11	3.2%	1.17 [0.27, 2.07]
Ögün2019	46.54	7.91	33	40.06	8.33	32	3.9%	0.79 [0.28, 1.29]
Patrícia2019	31.25	8.02	16	29.4	10.72	15	3.5%	0.19 [-0.51, 0.90]
Sungbae2024	37.26	11.22	15	38.8	12.13	15	3.5%	-0.13 [-0.84, 0.59]
**Subtotal (95% CI)**			**248**			**244**	**34.4%**	**1.12 [0.43, 1.80]**
Heterogeneity: Tau² = 1.06; Chi² = 101.24, df = 9 (P < 0.00001); I² = 91%								
Test for overall effect: Z = 3.19 (P = 0.001)								
**1.2.2 Semi-immersive virtual reality**								
Ain2021	52.2	10.69	25	35.36	12.73	25	3.7%	1.41 [0.79, 2.03]
Choi2014	40.3	18.4	10	47.2	10.5	10	3.2%	-0.44 [-1.33, 0.45]
Elena2021	40.6	19.4	15	41.8	21.4	14	3.5%	-0.06 [-0.79, 0.67]
Johnson2020	47.5	5.8	28	36.1	5.3	30	3.7%	2.03 [1.39, 2.67]
Sevgi2018	43.05	10.53	19	34.44	10.53	16	3.6%	0.80 [0.10, 1.49]
Sin2013	47.72	15.34	18	34.59	20.72	17	3.6%	0.71 [0.02, 1.39]
**Subtotal (95% CI)**			**115**			**112**	**21.2%**	**0.77 [0.07, 1.48]**
Heterogeneity: Tau² = 0.64; Chi² = 30.18, df = 5 (P < 0.0001); I² = 83%								
Test for overall effect: Z = 2.15 (P = 0.03)								
**1.2.3 None-immersive virtual reality**								
Ase2025	59.1	4.4	7	44.7	15.8	7	2.7%	1.16 [-0.00, 2.33]
Chen2022	31.05	6.91	18	29.89	8.24	18	3.6%	0.15 [-0.51, 0.80]
Kim2018	49.4	14.2	12	46.8	16	11	3.3%	0.17 [-0.65, 0.99]
Kong2016	32.8	18.2	35	29.2	17.5	35	3.9%	0.20 [-0.27, 0.67]
Kottink2014	45	14	8	41	18	10	3.1%	0.23 [-0.70, 1.17]
Lam2022	48.18	13.05	47	41.56	18.87	46	4.0%	0.41 [-0.01, 0.82]
Levin2012	47.3	11.9	6	44.9	11.7	6	2.7%	0.19 [-0.95, 1.32]
Norouzi-Gheidari2019	46.3	16.8	9	47.8	12.3	9	3.1%	-0.10 [-1.02, 0.83]
Oh2019	39.5	15.1	17	38.6	18.5	14	3.5%	0.05 [-0.66, 0.76]
Piron2009	53.6	7.7	18	49.5	4.8	18	3.6%	0.62 [-0.05, 1.30]
Shin2016	58.3	1.7	24	49.6	2.7	22	3.0%	3.83 [2.83, 4.83]
Shin2022	54.8	8.27	20	45.13	10.44	16	3.5%	1.02 [0.31, 1.72]
Turolla2013	48.2	15.2	263	44.1	17.3	113	4.3%	0.26 [0.04, 0.48]
**Subtotal (95% CI)**			**484**			**325**	**44.5%**	**0.57 [0.19, 0.94]**
Heterogeneity: Tau² = 0.32; Chi² = 55.44, df = 12 (P < 0.00001); I² = 78%								
Test for overall effect: Z = 2.97 (P = 0.003)								
**Total (95% CI)**			**847**			**681**	**100.0%**	**0.77 [0.46, 1.08]**
Heterogeneity: Tau² = 0.57; Chi² = 197.11, df = 28 (P < 0.00001); I² = 86%								
Test for overall effect: Z = 4.91 (P < 0.00001)								
Test for subgroup differences: Chi² = 1.96, df = 2 (P = 0.38), I² = 0%								

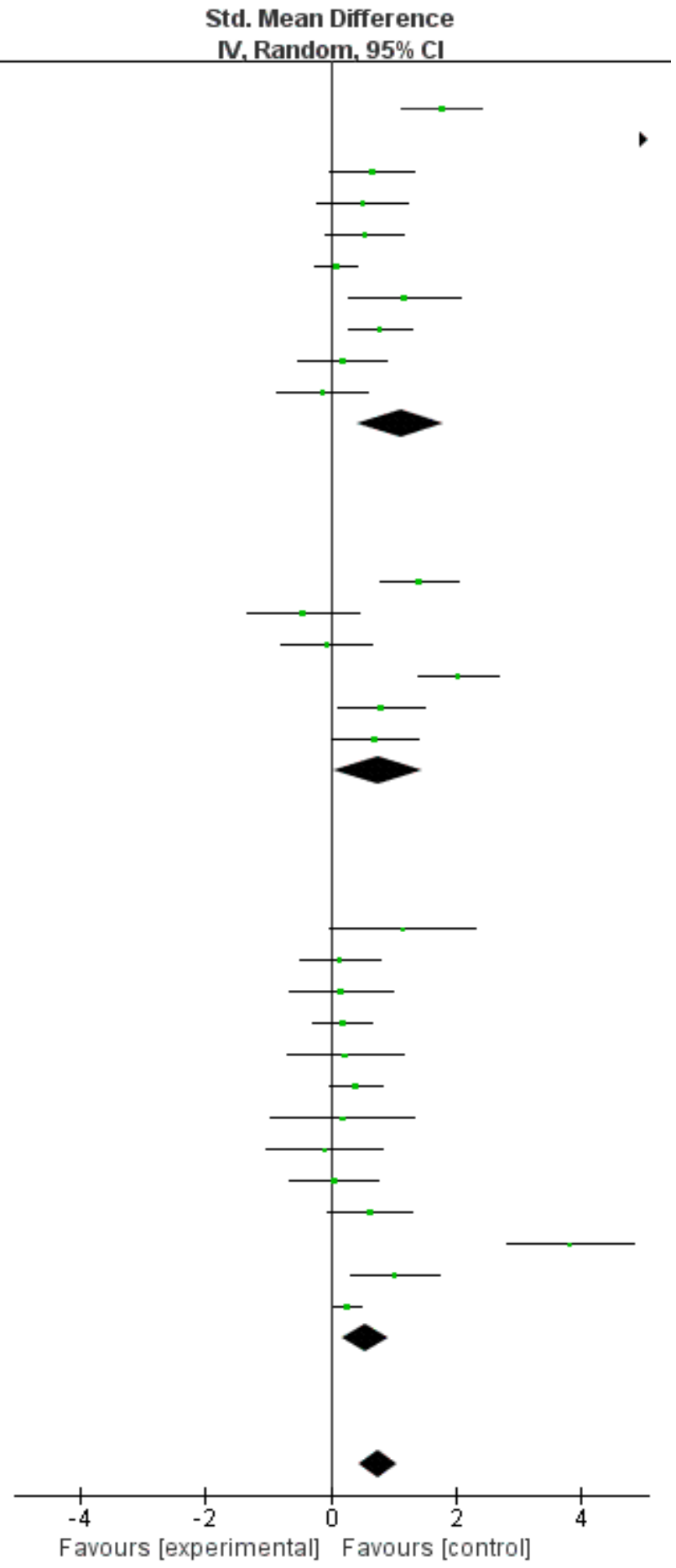

S4a. Different VR interventions VS Control (upper limb motor function)

Study or Subgroup	Experimental Mean	Experimental SD	Experimental Total	Control Mean	Control SD	Control Total	Weight	Std. Mean Difference IV, Random, 95% CI
**1.4.1 Chronic stroke**								
Ain2021	52.2	10.69	25	35.36	12.73	25	6.2%	1.41 [0.79, 2.03]
Elena2021	40.6	19.4	15	41.8	21.4	14	5.7%	-0.06 [-0.79, 0.67]
Hsu2022	46.1	13.7	18	35.1	18.6	17	6.0%	0.66 [-0.02, 1.34]
Huang2022	52.47	9.11	15	45.53	16.39	15	5.7%	0.51 [-0.22, 1.24]
Johnson2020	47.5	5.8	28	36.1	5.3	30	6.2%	2.03 [1.39, 2.67]
Patrícia2019	31.25	8.02	16	29.4	10.72	15	5.8%	0.19 [-0.51, 0.90]
Sin2013	47.72	15.34	18	34.59	20.72	17	5.9%	0.71 [0.02, 1.39]
Sungbae2024	37.26	11.22	15	38.8	12.13	15	5.8%	-0.13 [-0.84, 0.59]
**Subtotal (95% CI)**			**150**			**148**	**47.4%**	**0.68 [0.15, 1.21]**
Heterogeneity: Tau² = 0.45; Chi² = 32.91, df = 7 (P < 0.0001); I² = 79%								
Test for overall effect: Z = 2.52 (P = 0.01)								
**1.4.2 Subacute stroke**								
Amin2024	55.92	7.3	26	42.23	7.86	26	6.1%	1.78 [1.13, 2.43]
Chen2022	31.05	6.91	18	29.89	8.24	18	6.1%	0.15 [-0.51, 0.80]
Choi2014	40.3	18.4	10	47.2	10.5	10	5.0%	-0.44 [-1.33, 0.45]
Huang2024	34.9	18.4	20	24	20.7	20	6.2%	0.55 [-0.09, 1.18]
Kim2018	49.4	14.2	12	46.8	16	11	5.3%	0.17 [-0.65, 0.99]
Lam2022	48.18	13.05	47	41.56	18.87	46	7.3%	0.41 [-0.01, 0.82]
Mekbib2021	12.25	4.58	12	7.7	2.54	11	4.9%	1.17 [0.27, 2.07]
Sevgi2018	43.05	10.53	19	34.44	10.53	16	5.9%	0.80 [0.10, 1.49]
Shin2022	54.8	8.27	20	45.13	10.44	16	5.9%	1.02 [0.31, 1.72]
**Subtotal (95% CI)**			**184**			**174**	**52.6%**	**0.63 [0.24, 1.03]**
Heterogeneity: Tau² = 0.24; Chi² = 24.86, df = 8 (P = 0.002); I² = 68%								
Test for overall effect: Z = 3.12 (P = 0.002)								
**Total (95% CI)**			**334**			**322**	**100.0%**	**0.66 [0.34, 0.97]**
Heterogeneity: Tau² = 0.31; Chi² = 58.22, df = 16 (P < 0.00001); I² = 73%								
Test for overall effect: Z = 4.10 (P < 0.0001)								
Test for subgroup differences: Chi² = 0.02, df = 1 (P = 0.89), I² = 0%								

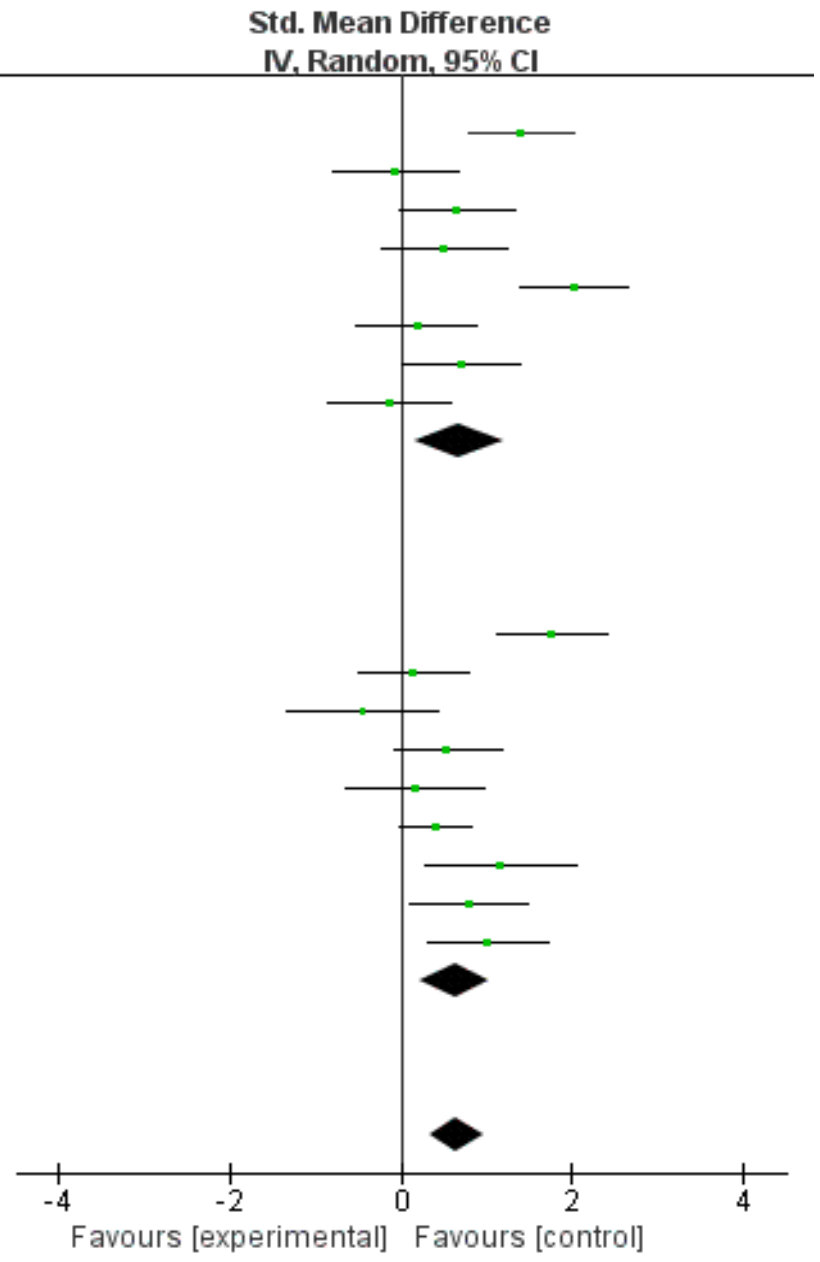

S4b. Subgroup analyses of different stroke subtypes (upper limb motor function)

Study or Subgroup	Experimental Mean	Experimental SD	Experimental Total	Control Mean	Control SD	Control Total	Weight	Std. Mean Difference IV, Random, 95% CI
**2.1.1 Immersive virtual reality**								
Amin2024	39.5	12.8	26	15.69	8.4	26	5.1%	2.17 [1.47, 2.86]
Hsu2022	22.6	19.5	18	14.9	20.9	17	5.2%	0.37 [-0.30, 1.04]
Ögün2019	41.15	7.82	33	32.09	5.94	32	5.7%	1.29 [0.75, 1.82]
Sungbae2024	2.66	1.75	15	1.6	1.63	15	5.0%	0.61 [-0.12, 1.34]
**Subtotal (95% CI)**			**92**			**90**	**21.1%**	**1.11 [0.37, 1.86]**
Heterogeneity: Tau² = 0.47; Chi² = 15.71, df = 3 (P = 0.001); I² = 81%								
Test for overall effect: Z = 2.92 (P = 0.004)								
**2.1.2 Semi-immersive virtual reality**								
Ain2021	40.64	13.03	25	31.2	20.82	25	5.6%	0.54 [-0.03, 1.10]
Choi2014	26.6	17.5	10	29.6	11	10	4.5%	-0.20 [-1.08, 0.68]
Johnson2020	35.5	6.9	28	30.4	8.4	30	5.7%	0.65 [0.12, 1.18]
Sevgi2018	28.53	11.15	19	20.81	10.03	16	5.2%	0.71 [0.02, 1.40]
Sin2013	20.67	14.38	18	16.29	11.7	17	5.2%	0.33 [-0.34, 0.99]
**Subtotal (95% CI)**			**100**			**98**	**26.2%**	**0.48 [0.20, 0.77]**
Heterogeneity: Tau² = 0.00; Chi² = 3.34, df = 4 (P = 0.50); I² = 0%								
Test for overall effect: Z = 3.33 (P = 0.0009)								
**2.1.3 None-immersive virtual reality**								
Ase2025	21.9	11.9	7	13	8	7	3.7%	0.82 [-0.29, 1.93]
Chen2022	23.61	6.88	18	22.44	6.37	18	5.3%	0.17 [-0.48, 0.83]
Kim2018	13.2	12.5	12	15.6	15.1	11	4.7%	-0.17 [-0.99, 0.65]
Kong2016	20.1	18.6	35	17.9	17.7	35	5.9%	0.12 [-0.35, 0.59]
Kottink2014	35	17	8	37	23	10	4.3%	-0.09 [-1.02, 0.84]
Lam2022	40.95	17.04	47	35.93	21.82	46	6.1%	0.25 [-0.15, 0.66]
Levin2012	30.3	21.2	6	23.9	21.2	6	3.6%	0.28 [-0.86, 1.42]
Norouzi-Gheidari2019	27.2	22.8	9	31.6	14.7	9	4.3%	-0.22 [-1.15, 0.71]
Oh2019	25.1	15.4	17	29.7	21.2	14	5.1%	-0.25 [-0.96, 0.46]
Shin2016	43.1	5.9	24	26.4	5.8	22	4.6%	2.80 [1.97, 3.64]
Shin2022	33.1	21.12	20	13.88	14.62	16	5.1%	1.01 [0.31, 1.72]
**Subtotal (95% CI)**			**203**			**194**	**52.7%**	**0.42 [-0.04, 0.88]**
Heterogeneity: Tau² = 0.45; Chi² = 45.46, df = 10 (P < 0.00001); I² = 78%								
Test for overall effect: Z = 1.77 (P = 0.08)								
**Total (95% CI)**			**395**			**382**	**100.0%**	**0.57 [0.25, 0.88]**
Heterogeneity: Tau² = 0.38; Chi² = 80.86, df = 19 (P < 0.00001); I² = 77%								
Test for overall effect: Z = 3.53 (P = 0.0004)								
Test for subgroup differences: Chi² = 2.65, df = 2 (P = 0.27), I² = 24.6%								

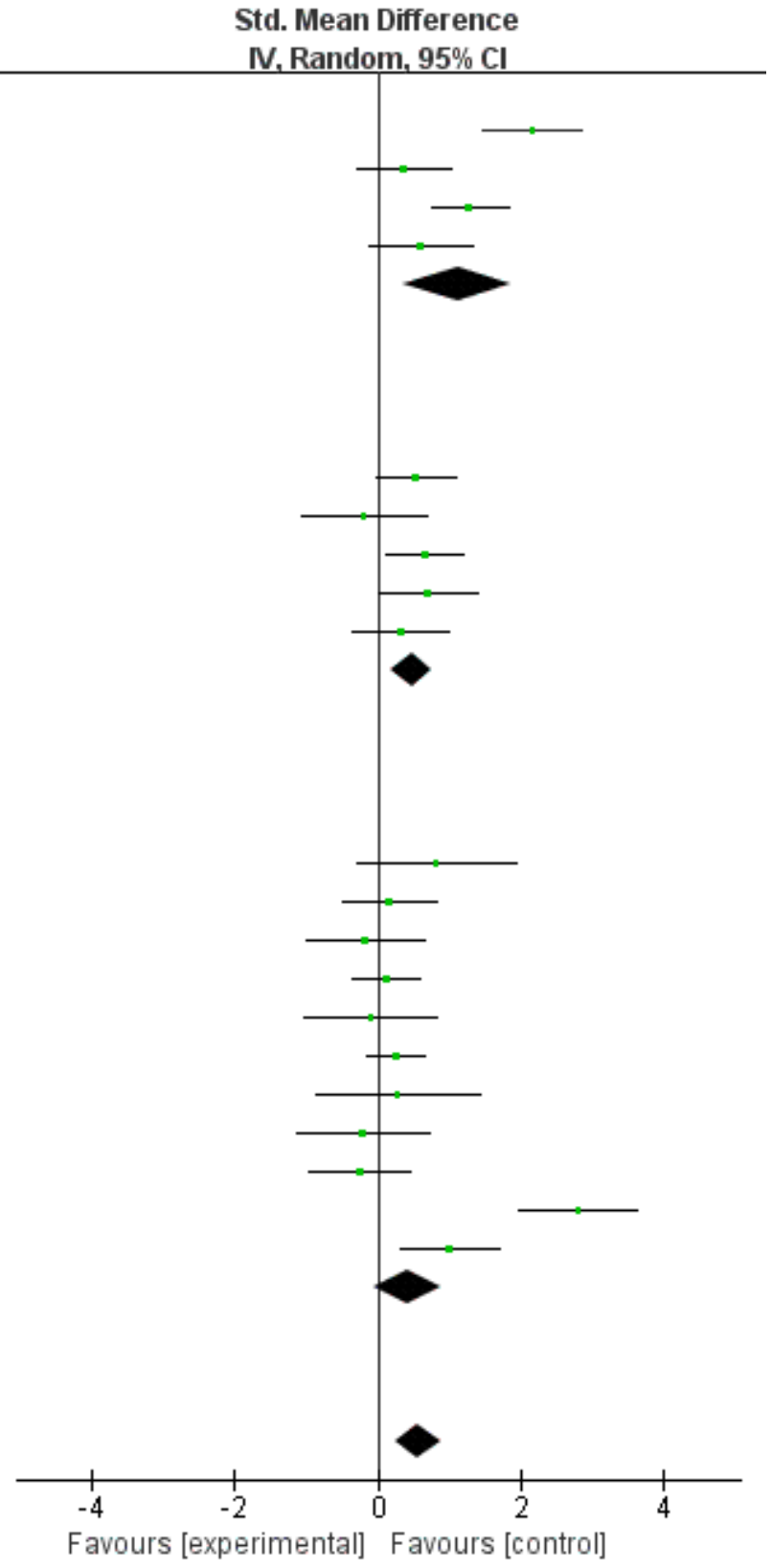

S4c. Different VR interventions VS Control (hand dexterity)

Study or Subgroup	Experimental Mean	Experimental SD	Experimental Total	Control Mean	Control SD	Control Total	Weight	Std. Mean Difference IV, Random, 95% CI
**2.2.1 Chronic stroke**								
Ain2021	40.64	13.03	25	31.2	20.82	25	9.2%	0.54 [-0.03, 1.10]
Hsu2022	22.6	19.5	18	14.9	20.9	17	8.3%	0.37 [-0.30, 1.04]
Johnson2020	35.5	6.9	28	30.4	8.4	30	9.6%	0.65 [0.12, 1.18]
Sin2013	20.67	14.38	18	16.29	11.7	17	8.3%	0.33 [-0.34, 0.99]
Sungbae2024	2.66	1.75	15	1.6	1.63	15	7.7%	0.61 [-0.12, 1.34]
**Subtotal (95% CI)**			**104**			**104**	**43.1%**	**0.51 [0.24, 0.79]**
Heterogeneity: Tau² = 0.00; Chi² = 0.81, df = 4 (P = 0.94); I² = 0%								
Test for overall effect: Z = 3.64 (P = 0.0003)								
**2.2.2 Subacute stroke**								
Amin2024	39.5	12.8	26	15.69	8.4	26	8.1%	2.17 [1.47, 2.86]
Chen2022	23.61	6.88	18	22.44	6.37	18	8.4%	0.17 [-0.48, 0.83]
Choi2014	26.6	17.5	10	29.6	11	10	6.6%	-0.20 [-1.08, 0.68]
Kim2018	13.2	12.5	12	15.6	15.1	11	7.0%	-0.17 [-0.99, 0.65]
Lam2022	40.95	17.04	47	35.93	21.82	46	10.7%	0.25 [-0.15, 0.66]
Sevgi2018	28.53	11.15	19	20.81	10.03	16	8.1%	0.71 [0.02, 1.40]
Shin2022	33.1	21.12	20	13.88	14.62	16	8.0%	1.01 [0.31, 1.72]
**Subtotal (95% CI)**			**152**			**143**	**56.9%**	**0.58 [-0.00, 1.15]**
Heterogeneity: Tau² = 0.48; Chi² = 31.65, df = 6 (P < 0.0001); I² = 81%								
Test for overall effect: Z = 1.96 (P = 0.05)								
**Total (95% CI)**			**256**			**247**	**100.0%**	**0.55 [0.23, 0.87]**
Heterogeneity: Tau² = 0.21; Chi² = 32.49, df = 11 (P = 0.0006); I² = 66%								
Test for overall effect: Z = 3.35 (P = 0.0008)								
Test for subgroup differences: Chi² = 0.04, df = 1 (P = 0.85), I² = 0%								

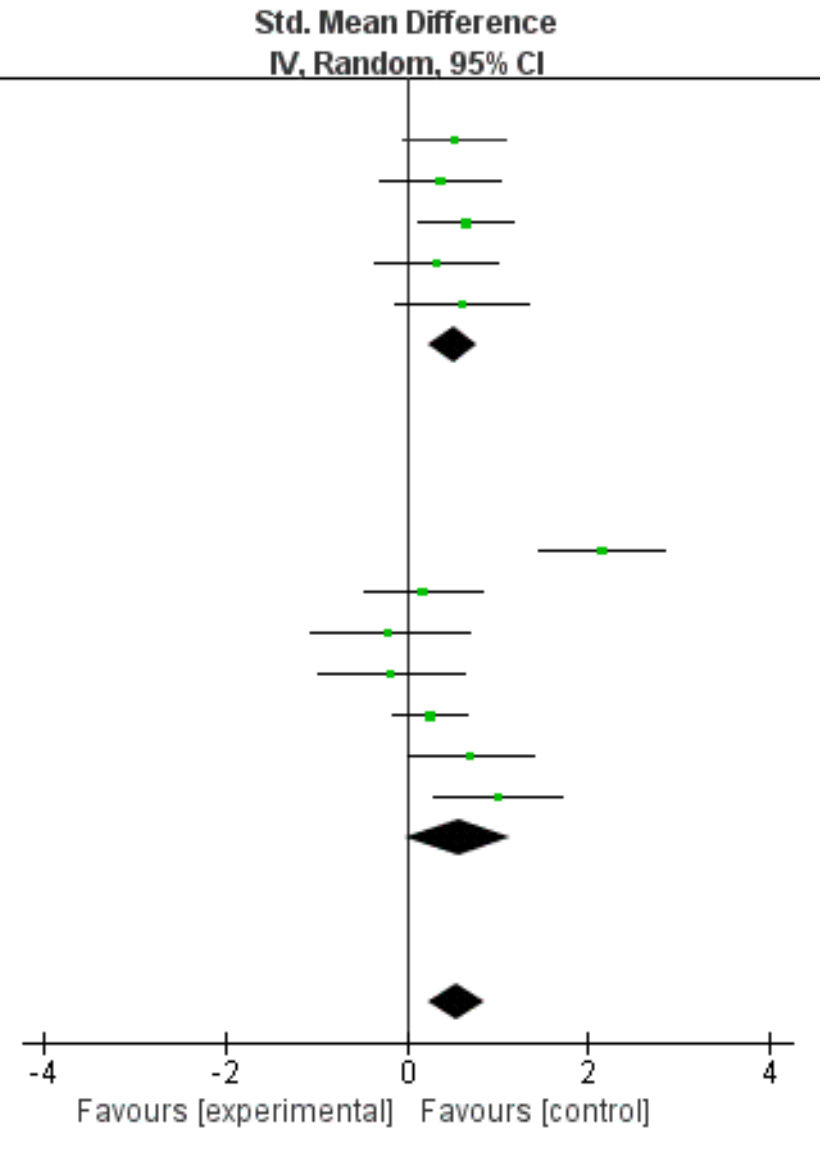

S4d. Subgroup analyses of different stroke subtypes (hand dexterity)

Study or Subgroup	Experimental Mean	Experimental SD	Experimental Total	Control Mean	Control SD	Control Total	Weight	Std. Mean Difference IV, Random, 95% CI
**3.1.1 Immersive virtual reality**								
Amin2024	16.26	2.3	26	10.57	2.84	26	8.2%	2.17 [1.47, 2.86]
Bai2022	78.32	1.32	25	72.51	1.52	25	6.8%	4.02 [3.03, 5.01]
Huang2024	85.3	12.2	20	72.8	21.7	20	8.5%	0.70 [0.06, 1.34]
Kiper2018	104.4	18.5	68	100.66	17.53	68	9.7%	0.21 [-0.13, 0.54]
Mekbib2021	32.27	6.84	12	28	7.15	11	7.5%	0.59 [-0.25, 1.43]
Ögün2019	89.6	8.2	33	84.96	6.42	32	9.1%	0.62 [0.12, 1.12]
**Subtotal (95% CI)**			**184**			**182**	**49.7%**	**1.32 [0.42, 2.23]**
Heterogeneity: Tau² = 1.14; Chi² = 68.17, df = 5 (P < 0.00001); I² = 93%								
Test for overall effect: Z = 2.88 (P = 0.004)								
**3.1.2 Semi-immersive virtual reality**								
Choi2014	85	11.6	10	86.9	10.5	10	7.3%	-0.16 [-1.04, 0.71]
Elena2021	85.3	11.8	15	75.7	10.7	14	7.9%	0.83 [0.06, 1.59]
Sevgi2018	23.74	4.42	19	23.63	4.99	16	8.3%	0.02 [-0.64, 0.69]
**Subtotal (95% CI)**			**44**			**40**	**23.5%**	**0.24 [-0.34, 0.82]**
Heterogeneity: Tau² = 0.11; Chi² = 3.49, df = 2 (P = 0.17); I² = 43%								
Test for overall effect: Z = 0.80 (P = 0.42)								
**3.1.3 None-immersive virtual reality**								
Kim2018	80.2	14.3	12	71.6	15.5	11	7.5%	0.56 [-0.28, 1.39]
Kong2016	87.6	18.5	35	91.3	17	35	9.2%	-0.21 [-0.68, 0.26]
Turolla2013	110.8	16.4	263	101.9	19.1	113	10.0%	0.51 [0.29, 0.74]
**Subtotal (95% CI)**			**310**			**159**	**26.7%**	**0.28 [-0.25, 0.80]**
Heterogeneity: Tau² = 0.15; Chi² = 7.53, df = 2 (P = 0.02); I² = 73%								
Test for overall effect: Z = 1.03 (P = 0.30)								
**Total (95% CI)**			**538**			**381**	**100.0%**	**0.76 [0.32, 1.20]**
Heterogeneity: Tau² = 0.49; Chi² = 87.60, df = 11 (P < 0.00001); I² = 87%								
Test for overall effect: Z = 3.37 (P = 0.0008)								
Test for subgroup differences: Chi² = 4.54, df = 2 (P = 0.10), I² = 55.9%								

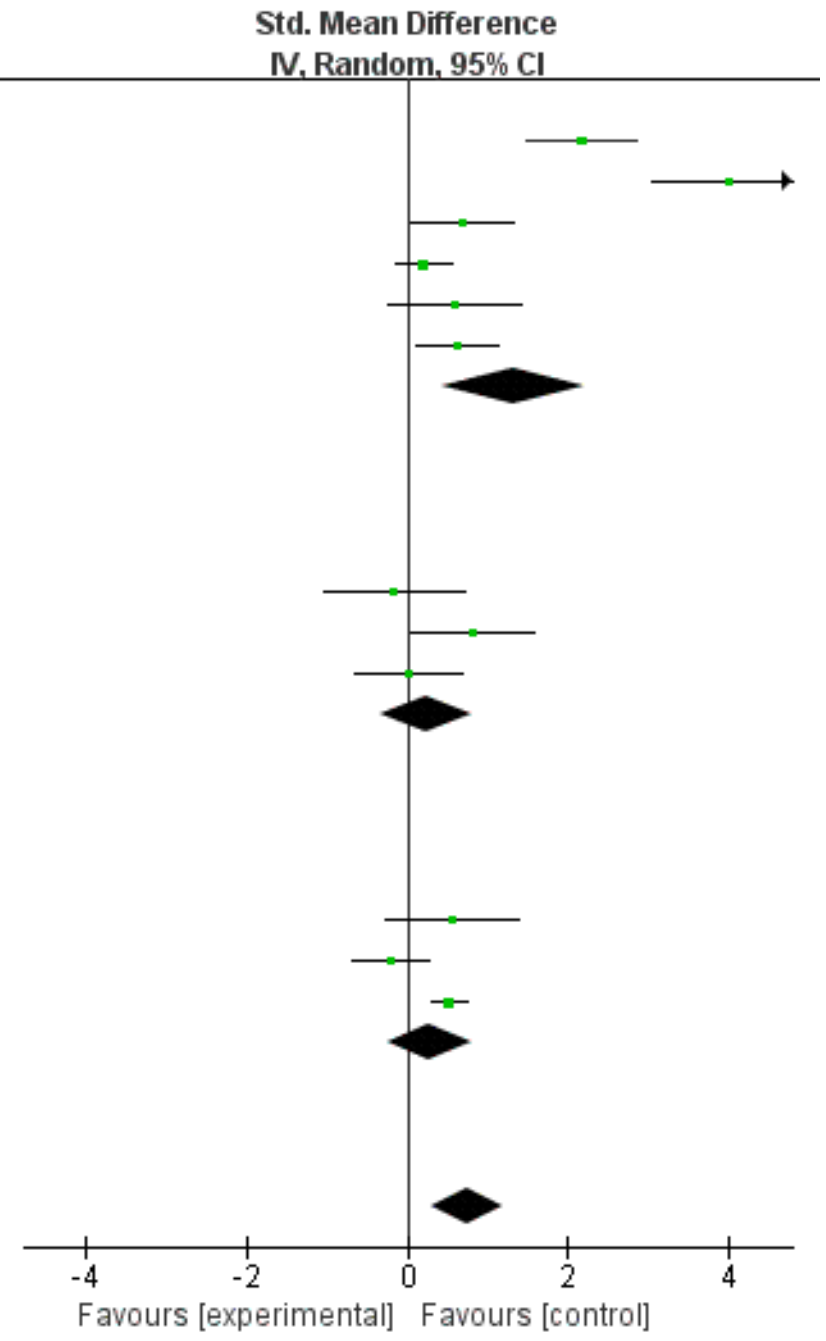

S4e. Different VR interventions VS Control (independence in daily life)

Study or Subgroup	Experimental Mean	Experimental SD	Experimental Total	Control Mean	Control SD	Control Total	Weight	Std. Mean Difference IV, Random, 95% CI
**3.2.1 Subacute stroke**								
Amin2024	16.26	2.3	26	10.57	2.84	26	14.9%	2.17 [1.47, 2.86]
Choi2014	85	11.6	10	86.9	10.5	10	13.2%	-0.16 [-1.04, 0.71]
Huang2024	85.3	12.2	20	72.8	21.7	20	15.4%	0.70 [0.06, 1.34]
Kim2018	80.2	14.3	12	71.6	15.5	11	13.6%	0.56 [-0.28, 1.39]
Mekbib2021	32.27	6.84	12	28	7.15	11	13.6%	0.59 [-0.25, 1.43]
Sevgi2018	23.74	4.42	19	23.63	4.99	16	15.2%	0.02 [-0.64, 0.69]
**Subtotal (95% CI)**			**99**			**94**	**85.7%**	**0.66 [-0.03, 1.34]**
Heterogeneity: Tau² = 0.58; Chi² = 25.02, df = 5 (P = 0.0001); I² = 80%								
Test for overall effect: Z = 1.89 (P = 0.06)								
**3.2.2 Chronic stroke**								
Elena2021	85.3	11.8	15	75.7	10.7	14	14.3%	0.83 [0.06, 1.59]
**Subtotal (95% CI)**			**15**			**14**	**14.3%**	**0.83 [0.06, 1.59]**
Heterogeneity: Not applicable								
Test for overall effect: Z = 2.12 (P = 0.03)								
**Total (95% CI)**			**114**			**108**	**100.0%**	**0.68 [0.10, 1.27]**
Heterogeneity: Tau² = 0.46; Chi² = 25.11, df = 6 (P = 0.0003); I² = 76%								
Test for overall effect: Z = 2.31 (P = 0.02)								
Test for subgroup differences: Chi² = 0.10, df = 1 (P = 0.75), I² = 0%								

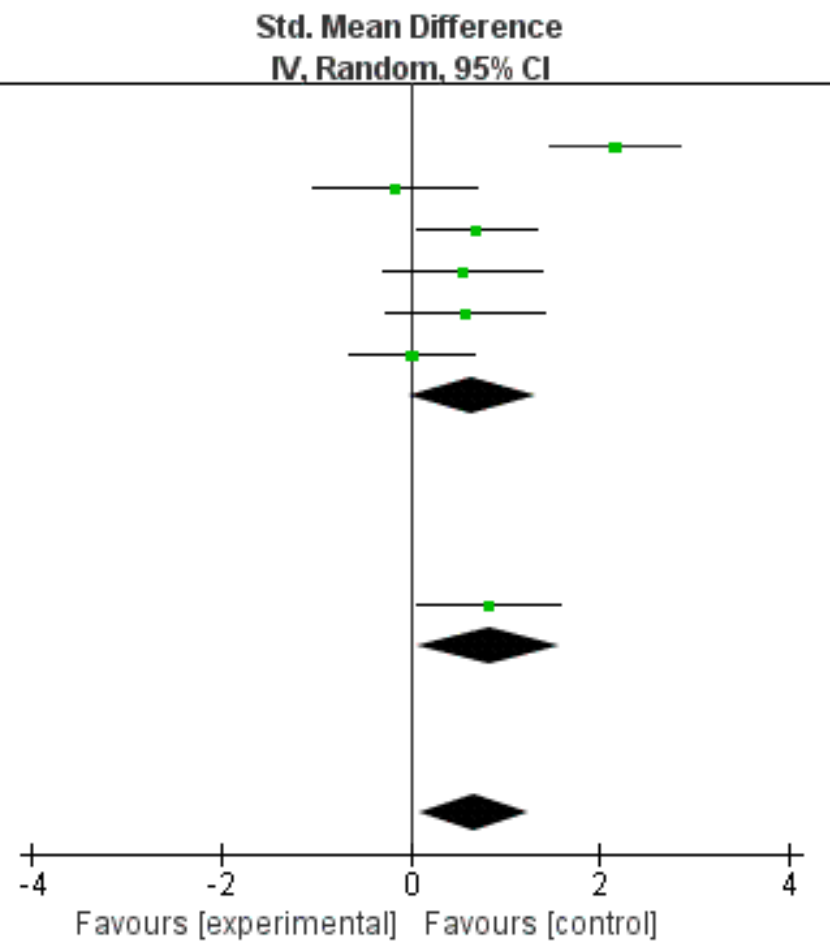

S4f. Subgroup analyses of different stroke subtypes (independence in daily life)

S5. The results of network meta-analysis related to upper limb motor function

```
Multivariate meta-analysis
Variance-covariance matrix = proportional .5*I(3)+.5*J(3,3,1)
Method = reml                                Number of dimensions     =      3
Restricted log likelihood = -134.75878       Number of observations   =     29
```

	Coef.	Std. Err.	z	P>|z|	[95% Conf.	Interval]
_y_B						
_cons	-.4434763	.7189612	-0.62	0.537	-1.852614	.9656617
_y_C						
_cons	-.5857242	.588356	-1.00	0.319	-1.738881	.5674324
_y_D						
_cons	-1.197388	.444338	-2.69	0.007	-2.068275	-.3265016

```
Estimated between-studies SDs and correlation matrix:
            SD        _y_B       _y_C       _y_D
_y_B  1.3358018          1          .          .
_y_C  1.3358018         .5          1          .
_y_D  1.3358018         .5         .5          1

Testing for inconsistency:
 ( 1)  - [_y_C]_cons + [_y_D]_cons = 0

           chi2(  1) =    2.50
         Prob > chi2 =    0.1139
```

S5a. Global inconsistency test

```
. network sidesplit all, tau

Side    Direct               Indirect              Difference
>   tau
        Coef.      Std. Err.  Coef.      Std. Err.  Coef.      Std. Err.  P>|z|
A D *   -1.376298   .5357891  -.3876801   8.174975  -.9886176   8.192508  0.904
>    1.538919
B D *   -.7502916   .6455979  -2.744168   25.86534   1.993876   25.87343  0.939
>    1.538654
C D *   -.7513498   .5341082  -2.710639   21.12811   1.959289   21.13484  0.926
>    1.538732
```

S5b. Local inconsistency test

Treatm~t	SUCRA	PrBest	MeanRank
IVR	86.1	67.2	1.4
SIVR	57.8	22.6	2.3
NIVR	50.9	10.2	2.5
Routine	5.1	0.0	3.8

S5c. Virtual reality relative ranking of upper limb motor function

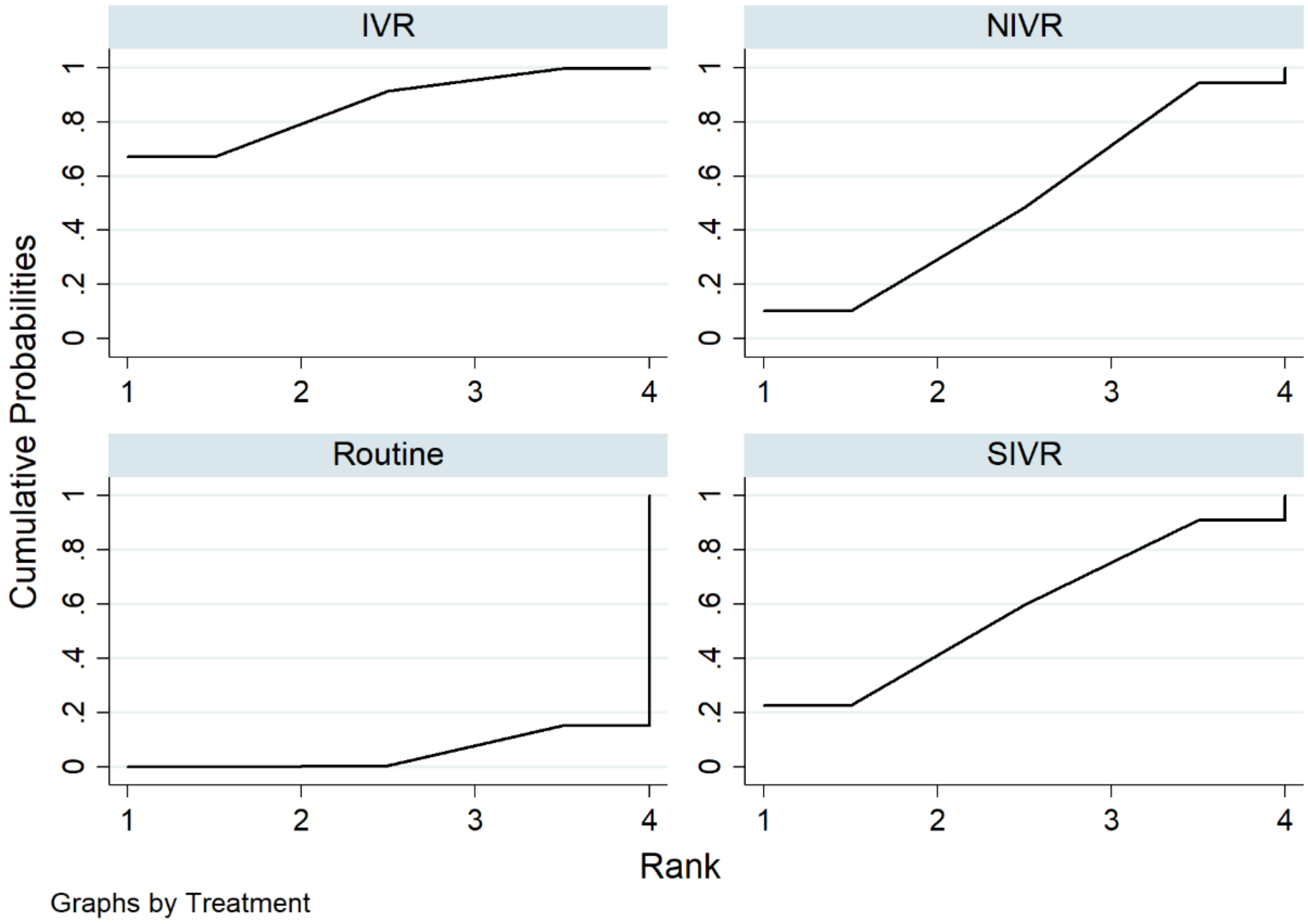

S5d. The upper limb motor function result of SUCRA

S6. The results of network meta-analysis related to hand dexterity

```
Meta-regression                                    Number of obs   =        20
REML estimate of between-study variance            tau2            =     .4167
% residual variation due to heterogeneity          I-squared_res   =    73.48%
Joint test for all covariates                      Model F(3,17)   =      4.61
With Knapp-Hartung modification                    Prob > F        =    0.0155
```

_y	Coef.	Std. Err.	t	P>|t|	[95% Conf.	Interval]
_trtdiff_B	-.6870012	.498436	-1.38	0.186	-1.738609	.3646069
_trtdiff_C	-.6961683	.4387059	-1.59	0.131	-1.621757	.2294203
_trtdiff_D	-1.113454	.371111	-3.00	0.008	-1.89643	-.3304778

```
Testing for inconsistency:
 ( 1)  - _trtdiff_C + _trtdiff_D = 0

       F(  1,    17) =    3.18
            Prob > F =    0.0924
```

S6a. Global inconsistency test

```
. network sidesplit all, tau

Side    Direct                Indirect              Difference
>   tau
        Coef.     Std. Err.  Coef.      Std. Err.  Coef.      Std. Err.  P>|z|
A D *   -1.113456  .3639281  -.2168259   7.90767   -.8966298   7.91604   0.910
>    .6453714
B D *   -.4264627  .3263663  -2.215558  28.29865   1.789095   28.30055   0.950
>    .6453369
C D *   -.4172786  .2294913  -2.21006   19.08628   1.792782   19.08767   0.925
>    .6453509
```

S6b. Local inconsistency test

Treatm~t	SUCRA	PrBest	MeanRank
IVR	95.6	89.0	1.1
SIVR	49.3	7.1	2.5
NIVR	50.5	3.9	2.5
Routine	4.5	0.0	3.9

S6c. Virtual reality relative ranking of hand dexterity

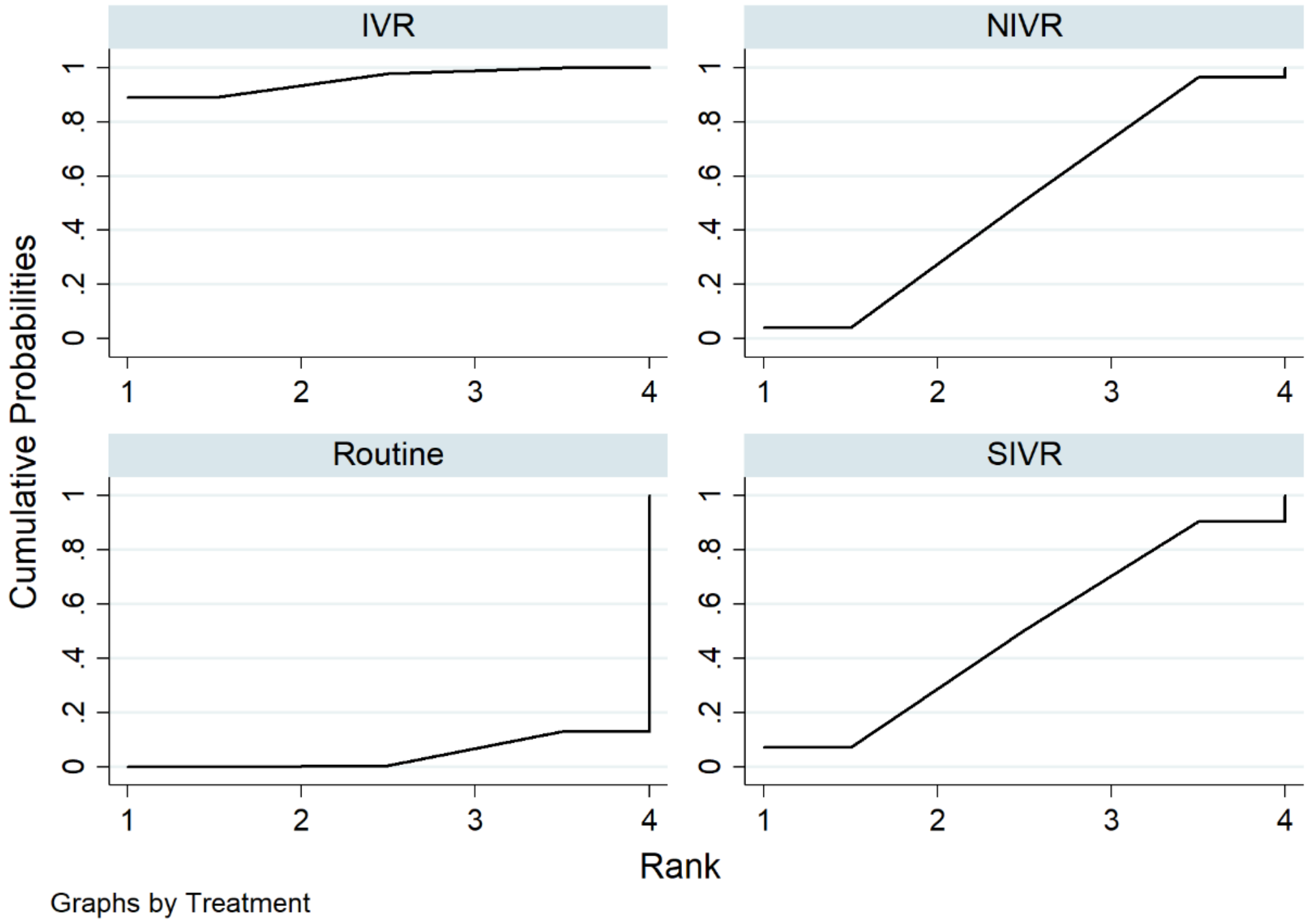

S6d. The hand dexterity result of SUCRA

S7. The results of network meta-analysis related to independence in daily life

```
Meta-regression                                    Number of obs  =        12
REML estimate of between-study variance            tau2           =     1.029
% residual variation due to heterogeneity          I-squared_res  =    88.65%
Joint test for all covariates                      Model F(3,9)   =      2.97
With Knapp-Hartung modification                    Prob > F       =    0.0893

------------------------------------------------------------------------------
          _y |      Coef.   Std. Err.      t    P>|t|     [95% Conf. Interval]
-------------+----------------------------------------------------------------
  _trtdiff_B |  -1.087464   .7865517    -1.38   0.200    -2.866768    .6918396
  _trtdiff_C |   -1.03659   .7695879    -1.35   0.211    -2.777519    .7043385
  _trtdiff_D |  -1.318708   .4499294    -2.93   0.017    -2.336519   -.3008974
------------------------------------------------------------------------------

Testing for inconsistency:
 ( 1)  - _trtdiff_C + _trtdiff_D = 0

       F(  1,     9) =    0.20
            Prob > F =    0.6621
```

S7a. Global inconsistency test

```
. network sidesplit all, tau

Side    Direct                Indirect              Difference
>   tau
        Coef.      Std. Err.  Coef.      Std. Err.  Coef.      Std. Err.  P>|z|
A D *   -1.318673   .4391525  -.1496894   12.91677  -1.168984   12.92423  0.928
>  1.014298
B D *   -.2312461   .6281428  -2.626155   36.54044   2.394909   36.54589  0.948
>   1.01423
C D *   -.2821143   .6078857  -2.560423   36.53977   2.278309   36.54486  0.950
>  1.014226
```

S7b. Local inconsistency test

Treatm~t	SUCRA	PrBest	MeanRank
IVR	94.9	86.1	1.2
SIVR	39.5	6.4	2.8
NIVR	43.1	7.5	2.7
Routine	22.5	0.0	3.3

S7c. Virtual reality relative ranking of independence in daily life

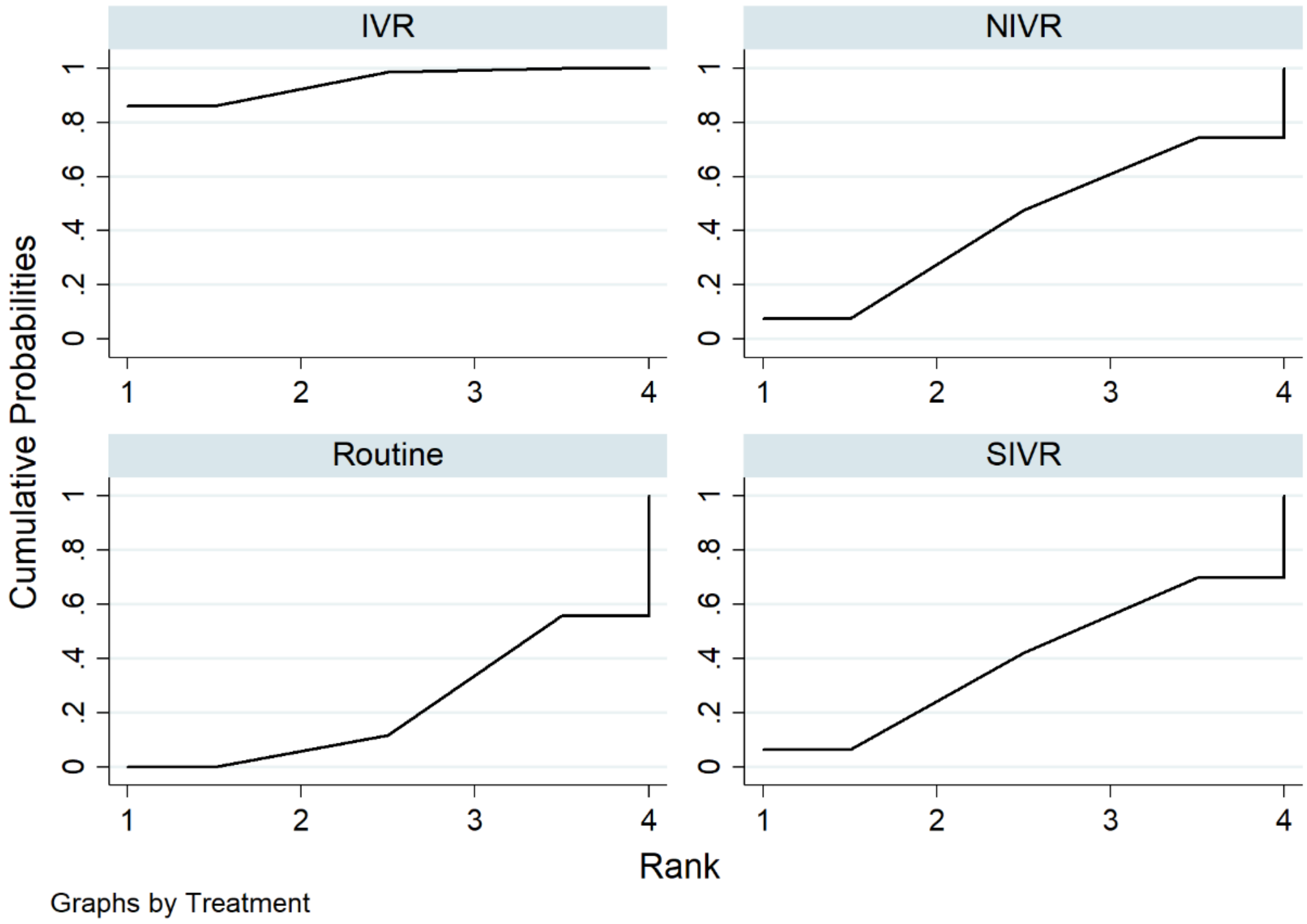

S7d. The independence in daily life result of SUCRA

S8. Funnel plot of all studies

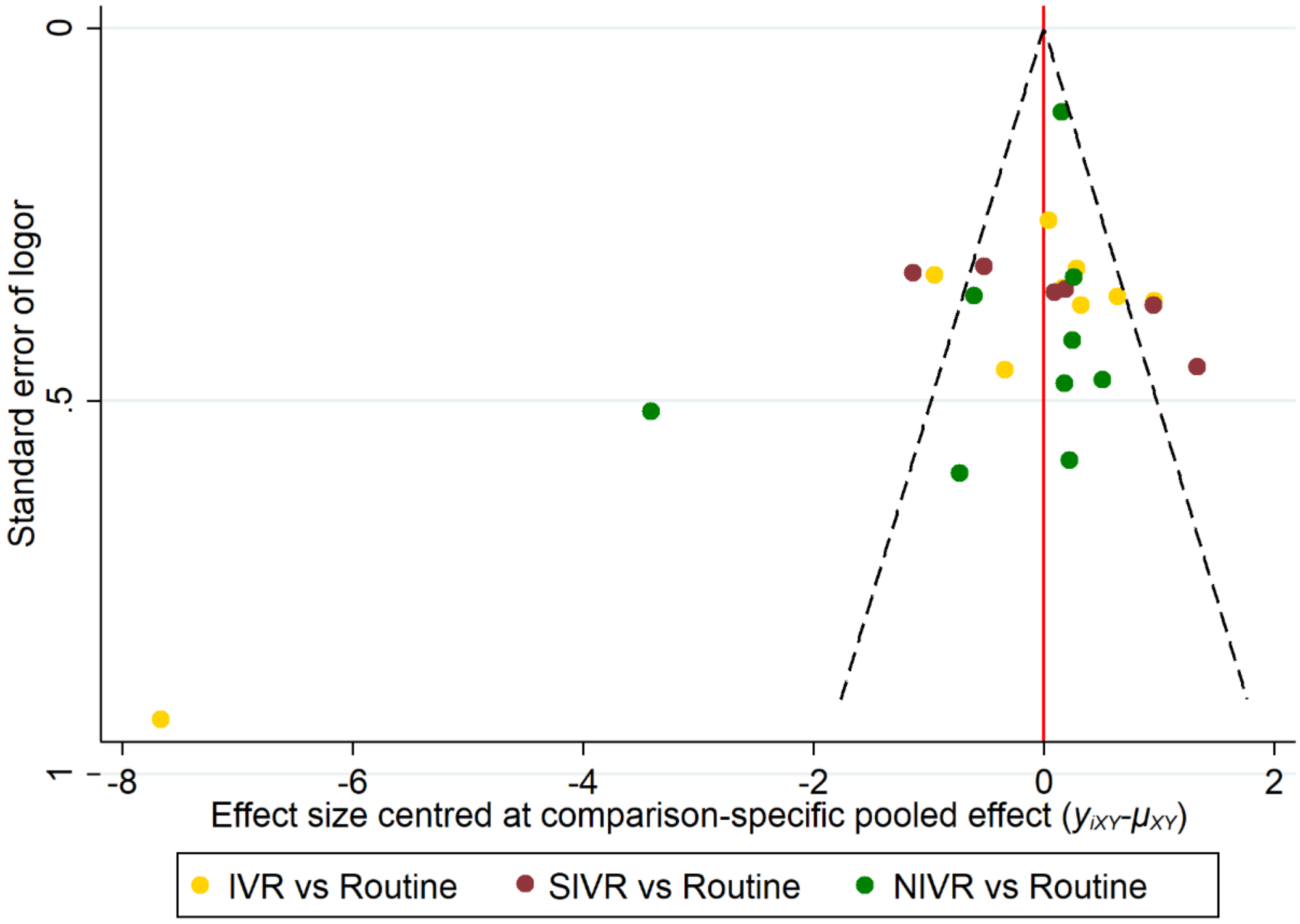

S8a.The comparison-adjusted funnel plot of upper limb motor function

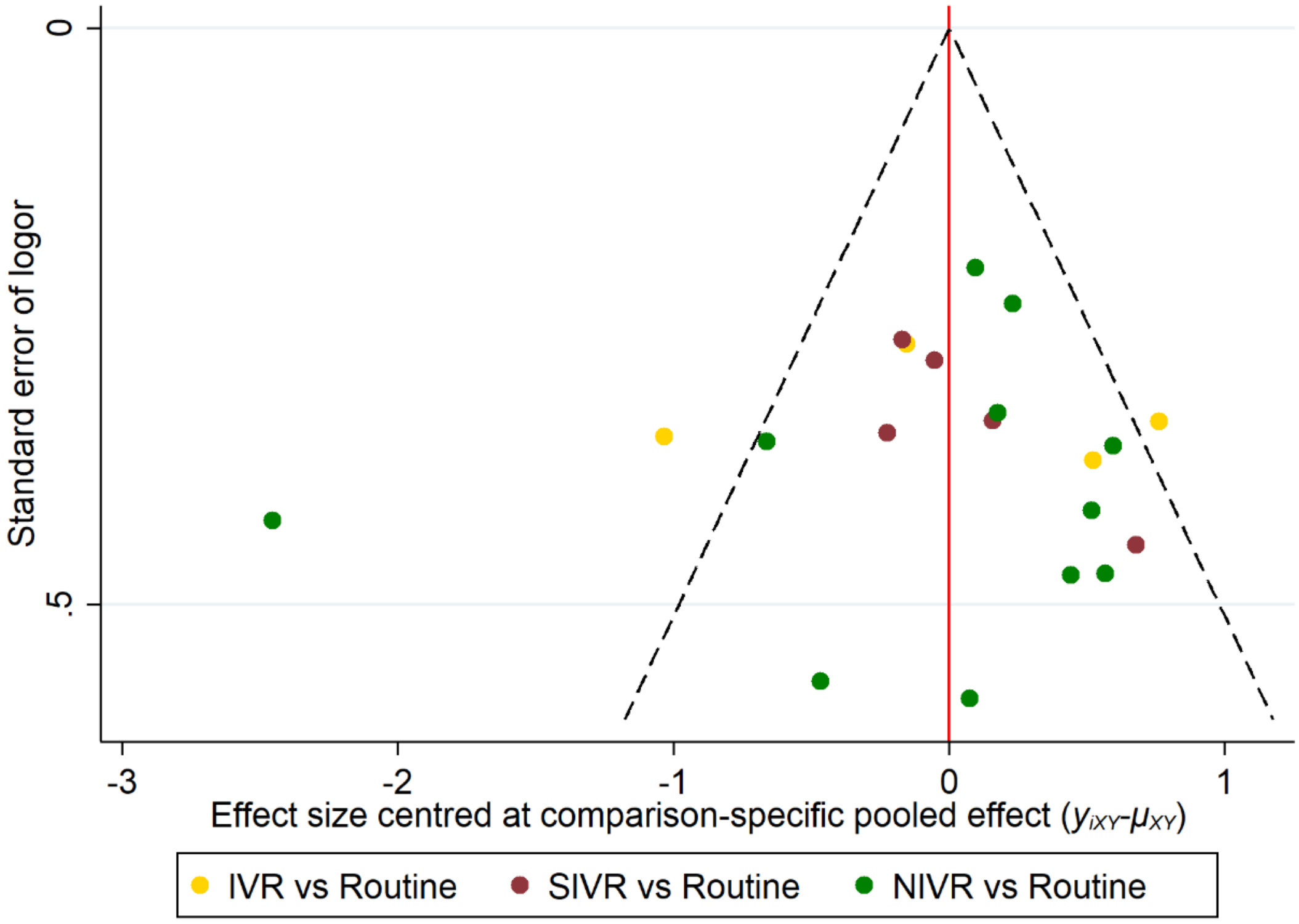

S8b.The comparison-adjusted funnel plot of hand dexterity

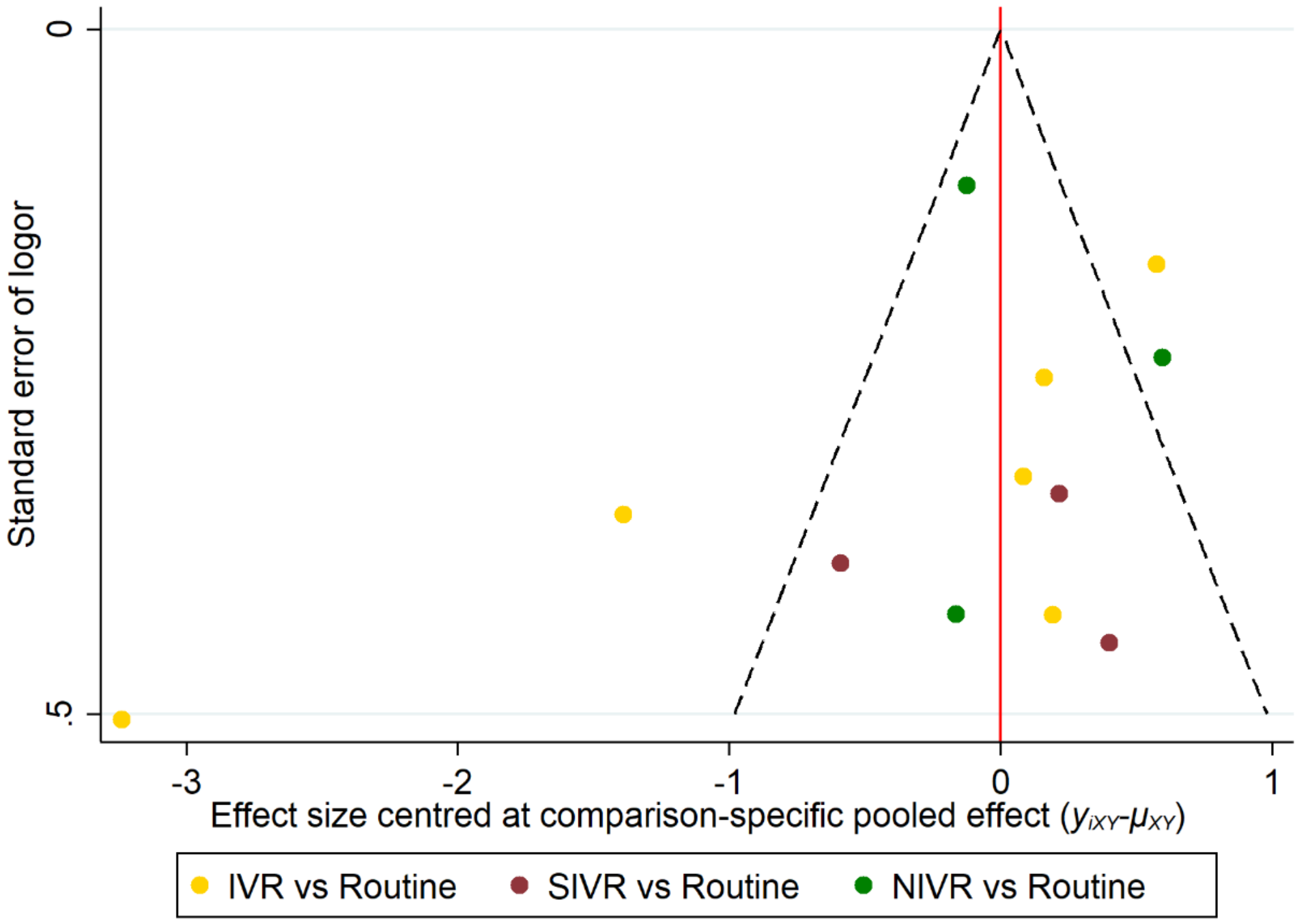

S8c.The comparison-adjusted funnel plot of independence in daily life

S9. Sensitivity Analysis Using Leave-one-out Method

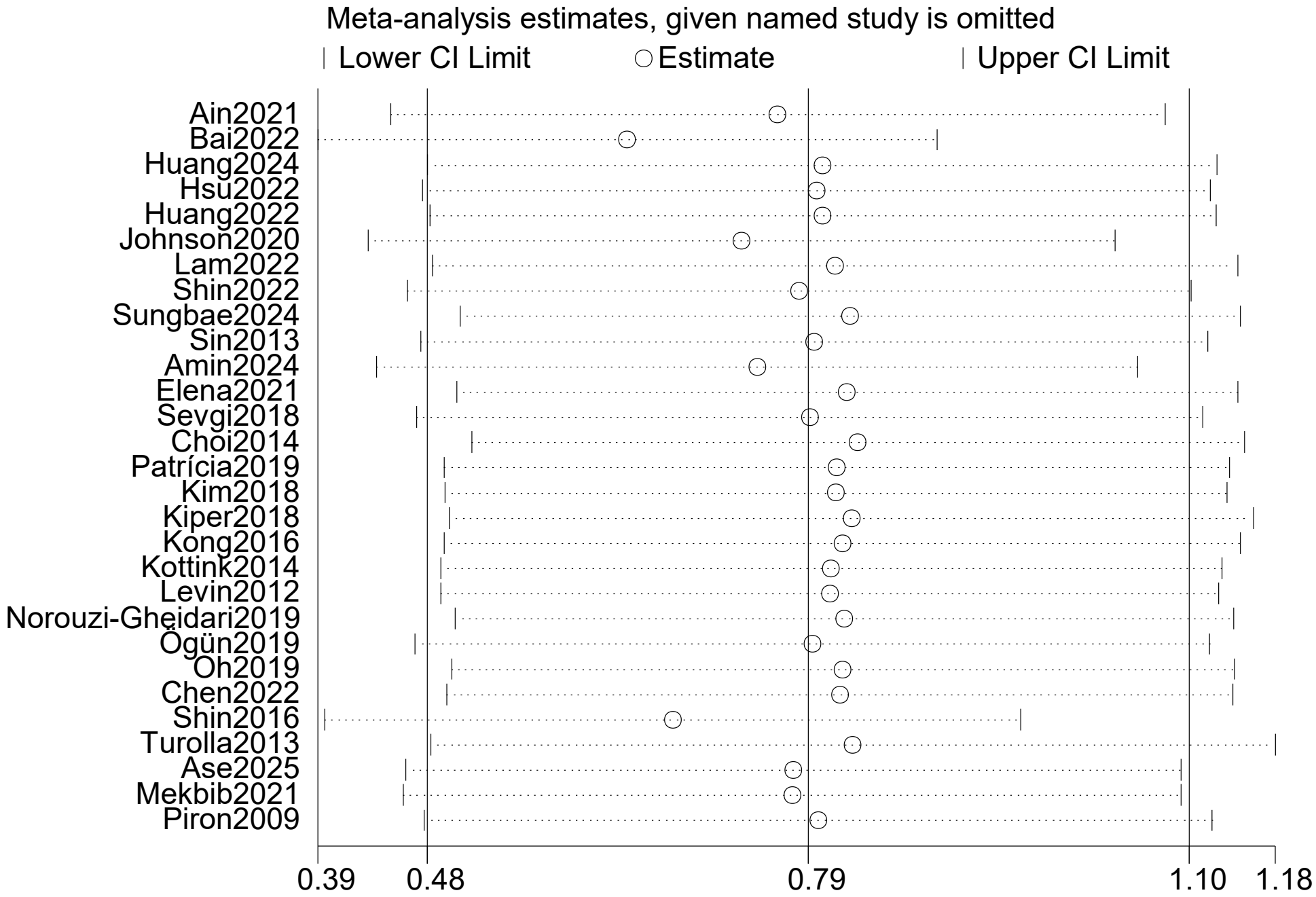

Supplement: Supplementary file 1 [file Data_Sheet_1.pdf]
